# CPE-Na-Based Hole Transport Layers for Improving the Stability in Nonfullerene Organic Solar Cells: A Comprehensive Study

**DOI:** 10.1021/acsami.4c01154

**Published:** 2024-03-25

**Authors:** Mohamed Samir, Enas Moustafa, Osbel Almora, Magaly Ramírez-Como, Maria Pilar Montero-Rama, José G. Sánchez, Emilio Palomares, Josep Pallarès, Lluis F. Marsal

**Affiliations:** † Department of Electronic, Electric and Automatic Engineering, Universitat Rovira i Virgili, Tarragona 43007, Spain; ‡ Science and Engineering of Renewable Energy Department, Faculty of Postgraduate Studies for Advanced Science, Beni Suef University, Beni Suef 62521, Egypt; § Sección de Estudios de Posgrado e Investigación, UPIITA Instituto Politécnico Nacional, Mexico City 07340, Mexico; ∥ Institute of Chemical Research of Catalonia-CERCA (ICIQ-CERCA), Tarragona 43007, Spain; ⊥ Institución Catalana de Investigación y Estudios Avanzados (ICREA), Barcelona 08010, Spain

**Keywords:** organic photovoltaics, nonfullerene acceptors, conjugated polyelectrolyte, hole transport layers, composite HTL, PCPDTPhSO3-Na (CPE-Na)

## Abstract

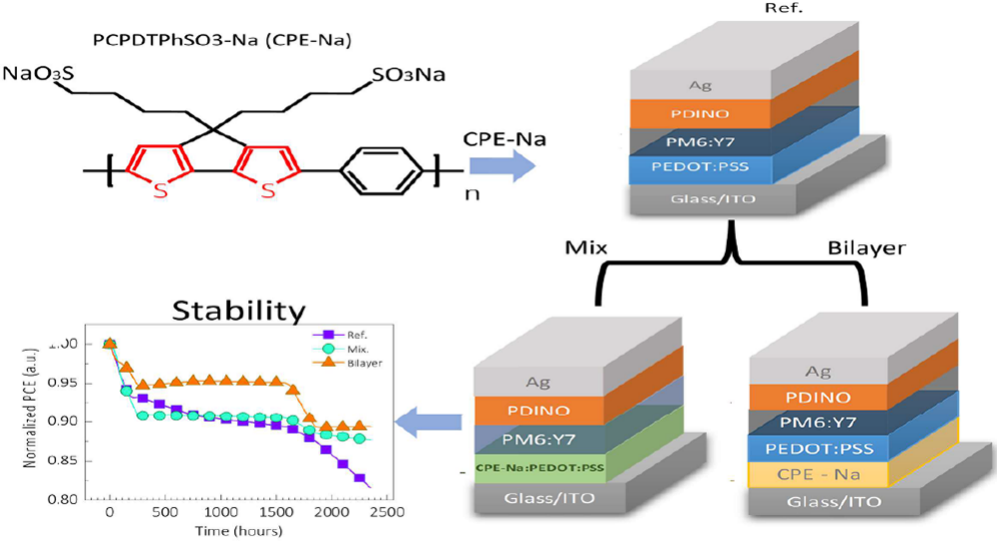

Organic photovoltaic (OPV) cells have experienced significant development in the last decades after the introduction of nonfullerene acceptor molecules with top power conversion efficiencies reported over 19% and considerable versatility, for example, with application in transparent/semitransparent and flexible photovoltaics. Yet, the optimization of the operational stability continues to be a challenge. This study presents a comprehensive investigation of the use of a conjugated polyelectrolyte polymer (CPE-Na) as a hole layer (HTL) to improve the performance and longevity of OPV cells. Two different fabrication approaches were adopted: integrating CPE-Na with PEDOT:PSS to create a composite HTL and using CPE-Na as a stand-alone bilayer deposited beneath PEDOT:PSS on the ITO substrate. These configurations were compared against a reference device employing PEDOT:PSS alone, as the HTL increased efficiency and fill factor. The instruments with CPE-Na also demonstrated increased stability in the dark and under simulated operational conditions. Device-based PEDOT:PSS as an HTL reached T80 after 2500 h while involving CPE-Na in the device kept at T90 in the same period, evidenced by a reduced degradation rate. Furthermore, the impedance spectroscopy and photoinduced transient methods suggest optimized charge transfer and reduced charge carrier recombination. These findings collectively highlight the potential of CPE-Na as a HTL optimizer material for nonfluorine OPV cells.

## 1 Introduction

From the first bulk heterojunction solar cells in the decade of 1990,^1^ through the introduction of small molecule and polymer nonfullerene acceptors (NFAs) in the decade 2000,^2^ the recent research progress in organic photovoltaic (OPV) devices has reported significant performance optimization^3^ with potential to become the cheapest form of electricity shortly.^4^ Not only have the latest single junction records surpassed 19% power conversion efficiency (PCE),^5−10^ but also significant improvements have been achieved among multijunction, flexible, and transparent/semitransparent solar cells.^3^ However, the long-term operational performance stability remains a challenge,^11−13^ and further device optimization research is undergoing for deploying the technology at an industrial scale fully.

**Figure 1:**
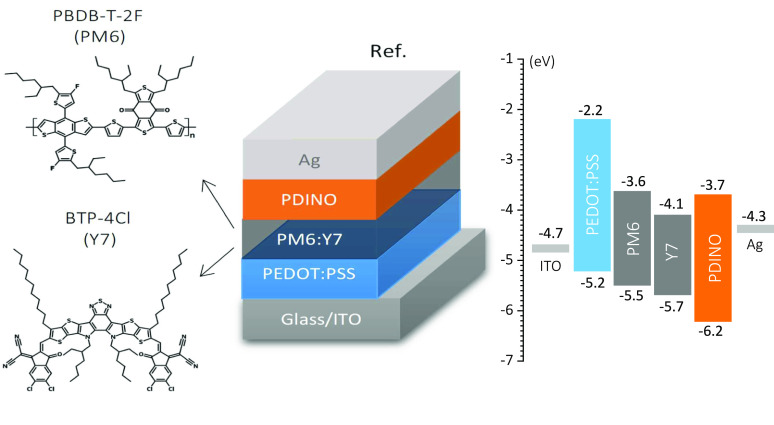
Schematic of the layer sequence structure of the reference (Ref.) sample with the corresponding approximate energy diagram before putting the materials in contact (right) and the PM6 and Y7 chemical structures (left). The literature values used for the energy positions in the energy diagram are summarized in Table S1.

Most typical optimization strategies for OPV devices comprise modifying at least one of the three main structural elements: the active absorption layer and the electron and hole transport layers, ETL and HTL, respectively. In this architecture, the active material is an organic semiconductor blend including at least a donor and an acceptor with a typical overall intrinsic (i) conductivity.^11^ Conversely, the ETL and HTL should be good conductors for only one type of charge carrier, i.e., n- and p-type semiconductors, respectively. Hence, a p–i–n or n–i–p device architecture can be obtained in a sequence of layers, most typically by using chemical deposition methods. Figure 1 shows the studied devices with a typical p–i–n structure, where the PEDOT:PSS layer is the p-type organic semiconductor, the blend (PM6:Y7) is the effective intrinsic (i) absorber, and the PDINO refers to the n-type selective layer. Moreover, the devices are completed with silver and indium–tin-oxide (ITO) electrodes on a glass substrate.

Among the active layer materials, the combined use of the donor PBDB-T-2F (PM6) and NFA BTP-4Cl (Y7) polymers has recently yielded PCE values above 17%^12,13^ and 18%^14^ for binary and ternary blends, respectively (see Figure 1, left). PM6 has been used in several studies reporting efficiencies above 19%.^5,6,8,9^ PM6 and Y7 are known for their excellent solubility in commonly used high-boiling halogenated solvents.^15^ Interestingly, Gao et al.^16^ reported the thermal stability of devices including this blend, which is enhanced by including an n-type conjugated polymer N2200 as the ternary component. In terms of versatility, Zheng et al.^17^ used the PM6:Y7 and Ag nanowires to fabricate semitransparent devices with a 19% average visible transmittance and a light utilization efficiency of 2.6%; Lee et al.^18^ have proved the fabrication of intrinsically stretchable OPV devices with this blend.

As an electron transport material partner for PM6-based active layers, the *N*,*N*′-bis(*N*,*N*-dimethylpropan-1-amine oxide)perylene-3,4,9,10-tetracarboxylic diimide (PDINO) has been successfully employed in recent studies.^19−22^ PDINO has been lovely for addressing the issue of air sensitivity and processing in green solvents.^23^ Moreover, its n-type self-doping effect^24^ and extrinsic doping viability^25^ are also favoring properties. Importantly, Moustafa et al.^21^ used PDINO as a low-temperature interface layer toward the ZnO ETL and reported improved device photostability.

The hole charge extraction, on the other hand, is often done with poly(3,4-ethylenedioxythiophene) polystyrenesulfonate (PEDOT:PSS), which is not only one of the most frequently used hole transport materials^26,27^ but also a successful one with several PCE reports above 19%.^8−10^ Nevertheless, the hygroscopic nature of PEDOT:PSS hinders the long-term stability of the device,^28,29^ and the acidity of PSS can lead to corrosion of some other layers in the cell, particularly the transparent conducting oxide contacts.^30−32^ In this context, exploring alternatives and additives for PEDOT:PSS-based HTLs in NFA OPV devices is suggested as a viable approach. Using two-dimensional MXene materials combined with PEDOT:PSS materials has recently enhanced the device’s performance—for instance, Liu et al.^33^ reported using Mo_1.33_C MXene, whereas Deng et al.^8^ optimized the HTL by including niobium-carbide (Nb_2_C) MXene, which was suggested to facilitate the phase separation of the PEDOT and PSS segments, thus improving the conductivity and work function of PEDOT:PSS.

Several studies have been reported on additives to enhance the HTL properties of PEDOT:PSS. For example, Zhou et al. recently correlated the change in the work function of PEDOT:PSS to the use of fluorine-containing surfactants and an increase in device performance. In addition, the combination of SnO_2_ and PEDOT:PSS has been proven to work as interconnected layers in inverted and conventional tandem polymer solar cells.^34^

Conjugated polyelectrolytes (CPEs) are distinguished by their organic semiconductor structure, complemented by ionic side chains that impart solubility in polar solvents.^35^ They share common traits with PEDOT:PSS, including high optical transmittance within the visible wavelength spectrum and high HOMO levels for hole transportation while maintaining low LUMO levels to block electrons. Their affinity with environmentally benign polar solvents such as alcohol and water facilitates effective dissolution, which in turn supports the creation of multilayer coatings through solution processing in organic optoelectronic apparatus.^36^ In addition, the ionic functionality inherent to CPEs aids in generating enduring surface dipoles, which modulate the work function of electrodes by creating interfacial dipoles at the juncture of the active layer and electrode.^37^ In 2018, Moon et al.^38^ demonstrated the CPE as HTL for P3HT-PCBM-based devices with PCE values of ∼3%. Despite their narrow bandgap, they reported that thin CPE films facilitate adequate light absorption within the active layer. The improved device efficiency is credited to the low surface roughness, high visibility region transmittance, and diminished charge transfer resistance.

This study demonstrates nonfullerene acceptor organic solar cells with an optimized hole transport layer using conjugated polyelectrolytes with device power conversion efficiency values >17%. We introduce two approaches for using PCPDTPhSO3-Na (CPE-Na): (i) as a standalone interlayer between the PEDOT:PSS and the ITO and (ii) as a mixture with the PEDOT:PSS. The first approach, herein called the Bilayer (CPE-Na/PEDOT:PSS), is shown as a promising electrode for high device stability by protecting the ITO from the acidity of PSS. Our study comprises a comparison with CPE-Na-free reference samples (see Figure 1) through a series of optoelectronic device characterization techniques, including photoinduced transient photovoltage (TPV), charge extraction (CE), and impedance spectroscopy (IS).

## 2 Experimental Section

### 2.1 Materials

The indium tin oxide (ITO) patterned glass substrate, having a resistivity of 10 Ω sq^–1^, was procured from PsiOTec. One-Material supplied several materials, which included PDINO 2,9-bis[3-(dimethyloxidoamino)propyl]anthra[2,1,9-*def*:6,5,10-*d*’*e*’*f*’]diisoquinoline1,3,8,10(2*H*,9*H*)-tetrone, PM6 polymer donor (poly[2,6-(4,8-bis(5-(2-ethylhexyl-3-fluoro)thiophen-2-yl)benzo[1,2-*b*;4,5-b′]dithiophene))-*alt*-(5,5-(1′,3′-di-2-thienyl-5′,7′-bis(2-ethylhexyl)benzo[1′,2′-*c*:4′,5′-*c*’]dithiophene-4,8-dione)]) (PBDB-T-2F), and Y7 nonfullerene acceptor (2,2′-((2*Z*,2′*Z*)-((12,13-bis(2-ethylhexyl)-3,9-diundecyl-12,13-dihydro-[1,2,5]thiadiazolo[3,4-*e*]thieno[2’,’3′:’4′,5′]thieno[2′,3′:4,5] pyrrolo[3,2-g]thieno[2′,3′:4,5]thieno[3,2-*b*]indole-2,10-diyl)bis(methanylylidene))bis(5,6-dicholoro-3-oxo-2,3-dihydro-1*H*-indene-2,1-diylidene))dimalononitrile) (BTP-4Cl).

Clevios P VP Al 4083 from H.C. Starck provided the PEDOT:PSS aqueous solution, and Testbourne Ltd. furnished the 99.999% pure silver (Ag). Methanol, chlorobenzene (CB), and chloronaphthalene (CN) were all sourced from Sigma-Aldrich. The conjugated poly electrolyte material PCPDTPhSO3-Na (CPEPh-Na) was supplied from One-Material. Through this procurement, we managed to secure the necessary high-quality materials required for our research.

### 2.2 Device Fabrication

Initially, detergent and water were used to ultrasonicate the ITO substrates, which was subsequently followed by a 10 min ultrasonication in ethanol, methanol, and isopropanol. Post this treatment, the ITOs were places in an oven set at a temperature of 100 °C for a duration of 10 min. To conclude the process, the samples were exposed to UV-ozone treatment for a period of 20 min. This final step was crucial in eliminating any lingering organic residue and simultaneously activating the surfaces of the ITOs.

The PCPDTPhSO3 -Na (CPEPh-Na) solution was prepared by dissolving 0.1 mg in 1 mL of methanol and then filtered by 0.45 μm PFTE. The prepared solution was deposited over the cleaned ITO at 3000 rpm for 40 s in the bilayer-CPE-Na structure.

The PEDOT:PSS aqueous solution was first passed through a 0.45 μm PFTE (poly tetrafluoroethylene) filter. This filtered solution was then applied to the precleaned ITOs using a spin-coating technique. Following the coating process, the PEDOT:PSS film was subjected to an annealing process at a temperature of 150 °C for a span of 10 min in an air atmosphere. For the mixed layer, the deposition of the solution followed the same method as that for the deposition process of PEDOT:PSS.

A blend solution, composed of PM6 donor and Y7 acceptor, was prepared at a concentration of 20 mg/mL. This solution was achieved by dissolving these materials in a 1:1 ratio in CB, supplemented with 0.5 wt % CN solvent additive. This mixture was stirred consistently for a minimum duration of 3 h at a temperature of 80 °C. Following this, the prepared solution was filtered using a 0.2 μm PVDF filter. The filtered solution was then spin-coated directly onto the HTL at a speed of 2000 rpm, which lasted for a duration of 40 s. After the spin-coating process, a thermal treatment was conducted at a temperature of 90 °C.

PDINO solutions were created by dissolving PDINO at a concentration of 1.5 mg/mL in methanol. This solution was then filtered and subsequently spin-coated at a rate of 3000 rpm for a duration of 30 s, with no thermal annealing process undertaken. To conclude the fabrication process, a 100 nm thin film of Ag was thermally evaporated to serve as the top contact. This was performed under high vacuum conditions of less than or equal to 1 × 10^–6^ mbar, utilizing a shadow mask to define the active device area of 0.09 cm^2^.

### 2.3 Characterization

The current density–voltage (*J–V*) characteristics of the fabricated devices were conducted using a Keithley 2400 source-measure unit and a solar simulator from Abet Technology (model 11000 class type A, Xenon arc), both operated under standard room temperature conditions. The samples were mounted inside the N_2_-atmosphere globe box and kept in an isolating holder during measurement for preventing humidity-related degradation.

The EQE spectra were recorded from 300 to 1100 nm using Lasing IPCE-DC model equipment with the series number LS1109-232. The photoluminescence (PL) measurements were performed on a Fluorolog Horiba Jobin Yvon spectrofluorometer.

The absorbance spectra were carried on by using a PerkinElmer Lambda 950-UV–vis/NIR spectrometer at room temperature.The photoinduced charge extraction (CE) measurements were carried out in open-circuit voltage equilibrium by illuminating the devices using a white light LED ring from LUXEON Lumileds. The LED ring is connected to a power supply to control the applied bias, providing different light intensities. Once the *V*_OC_ is completely stabilized, the light is turned off, and the circuit is closed to force the charge to pass through an external circuit with a 50 Ohm resistance in parallel to an oscilloscope Yokogawa DLM2052. The voltage drop across the resistance is recorded by the oscilloscope.

The impedance spectroscopy measurements were carried out with a HP 4192A LF impedance analyzer. The homemade degradation chamber included an Helieon white LED, an RS PRO RS-3005P power source, and a BK precision 2831E multimeter. The temperature of the chamber was monitored with an Arduino controlled DS18B20 temperature sensor.

Morphology and topography images were obtained via scanning electron microscopy (SEM) and atomic force microscopy (AFM) in a tapping mode using silicon probes with a spring constant of 1–5 N m–^1^ and a resonant frequency of 75 kHz. Surface microstructure images were derived from a field emission scanning electron (FESEM) microscope, Thermo Fisher Scientific-Scico2-High Resolution.

## 3 Results and Discussion

### 3.1 Fabrication and Material Characterization

The mixed solution was fabricated by blending a PEDOT:PSS solution with a methanolic solution of CPE-Na. The concentration of CPE-Na in the methanol solution was maintained at 0.1 mg/mL. Both resolutions were combined in a predetermined ratio of (4:1), with PEDOT:PSS being four parts and CPE-Na being one part. This proportional blending was performed under controlled room temperature conditions. To ensure homogeneous mixing and proper interaction between the two solutions, the mixture was stirred continuously for 2 h. The stirring was executed at a consistent speed to maintain the equilibrium of the solution and to avert any potential discrepancies in the concentration.

**Figure 2:**
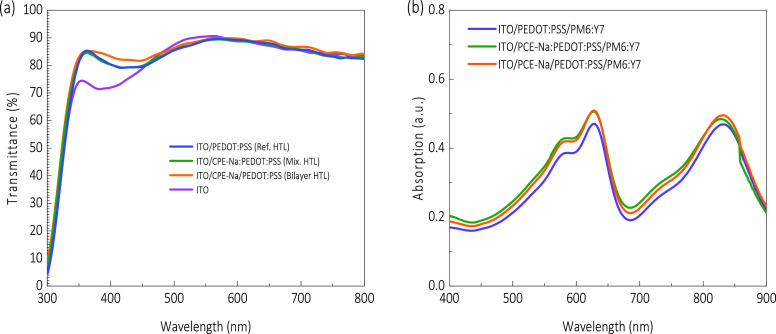
Optical (a) transmittance spectra of the different HTLs and corresponding (b) absorption after deposition of the absorber layer on top of the HTLs, as indicated.

The studied HTLs were morphologically and optically characterized and showed similar features. Figure 2a shows a comparable transmittance of ∼90% in the visible spectrum range, slightly more significant for the bilayer HTL concerning the reference and mixed alternatives of HTL. Even though a buffer layer of CPE-Na has been added between the ITO and the HTL, it does not alter the HTL’s transparency. Consequently, light harvesting for the active layer is maintained, as depicted in Figure 2b. Figure S1 shows no apparent difference between the scanning electron microscopy images, and the macroscopic photographs in Supporting Information for the three studied HTL sequences show no apparent difference. Subsequently, the contact angle technique was utilized for assessing the compatibility between the active layer solution and the three HTL alternatives, resulting in a good match, as presented in Figure S2, whose average angles are summarized in Table S2. The introduction of CPE-Na to the HTL, as either a mixed layer or bilayer, decreases the contact angle of the active layer, thereby promoting the spreading and adhesion of PM6:Y7 on the HTL. Table S2 provides each device’s contact angle measurement values, showing 16.7°, 14.2°, and 13.3° for ref-PEDOT:PSS, bilayer-CPE-Na, and mix-CPE-Na, respectively. Consequently, this leads to an improved interface between the two layers: the active layer and the HTL. Accordingly, the active layer PM6:Y7 was deposited on top of the three HTL variants, resulting in slightly increased absorption for the CPE-Na-including samples, as shown in Figure 2b.

For additional investigation, AFM topography and 3D images of the PM6:Y7 active layer have been introduced in the Figure S3a, where the RMS values for the active layer over the HTLs exhibit slight changes to 0.970 1.003, and 1.125 nm for the bilayer-CPE-Na, mix-CPE-Na, and ref-PEDOT:PSS, respectively. This decrease in roughness can be attributed to the changing contact angle of the active layer on the HTLs, which in turn affects the morphology of the active layer. We hypothesize that the modified contact angle influences the wetting behavior and interfacial interactions within the active layer, leading to smoother morphology.^39^ To investigate the efficiency of charge extraction in the fabricated hole transport layer (HTL), photoluminescence (PL) spectra were gathered from several films. These films comprised ITO/PM6:Y7, ITO/PEDOT:PSS/PM6:Y7, ITO/CPE-Na/PEDOT:PSS/PM6:Y7, and ITO/mix-CPE-Na/PM6:Y7, as illustrated in Figure S3b. The samples were excited at a wavelength of 635 nm. Notably, the mix-CPE-Na layer exhibited significant quenching compared to the other two structures. This improvement enhances the HTL’s ability to effectively extract hole carriers from the active layer, an achievement made possible by reducing the luminescence spectrum.^39^ The PL spectra in Figure S3b show several peaks, which can be associated with the donor PM6 or the acceptor Y7, as summarized in the literature summery of Table S3.^12^ The reduced photoluminescence recombination of the photogenerated carriers can be ascribed to the superior extraction ability of the carriers from the active blend. This is due to the improved film morphology of the active layer, as AFM images, which decreases the surface trap states existing on the layer interfaces.^40^ The quenching observed in the photoluminescence spectrum, particularly for the mix-CPE-Na layer, signifies a reduction in carrier recombination and supports the enhanced charge extraction capability of the hole transport layers (HTLs), and this may be because of the direct contact between the active layer and the CPE-Na molecules in the mixed HTL. Following Mihailetchi’s method (see Section S2.2),^41^ the photocurrent density (*J*_ph_) was measured about the effective applied voltage (*V*_eff_), resulting as in Figure S3c.

**Figure 3:**
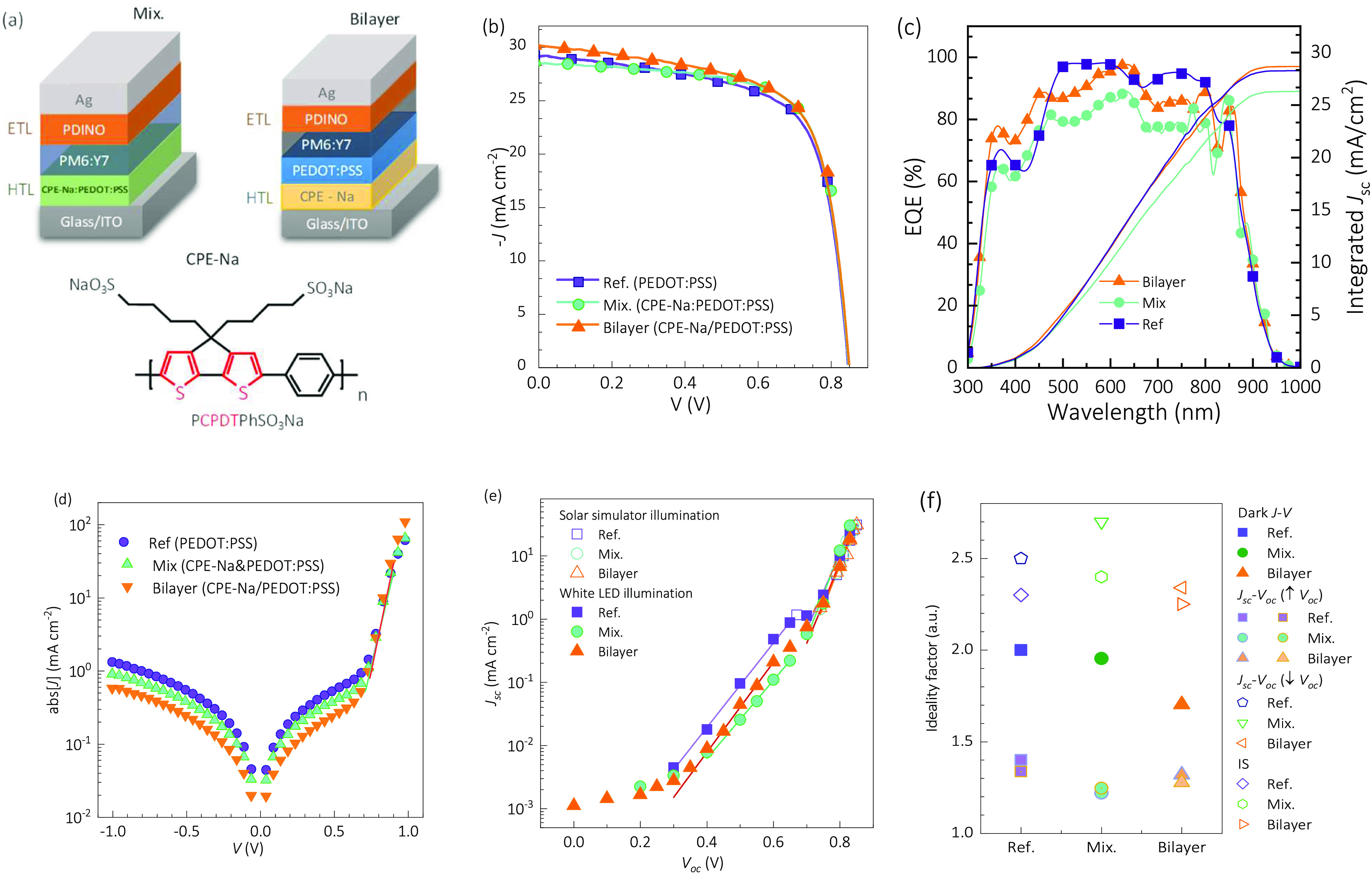
(a) Schemed structure of the studied devices and optoelectronic characterization: illustrative (b) current density–voltage measured with solar simulator; (c) external quantum efficiency; (d) dark current–voltage curves; (e) short-circuit current versus open-circuit current for different illumination conditions, as indicated; and (f) summary of ideality factors.

Three device structures were fabricated after the PDINO ETL deposition and the silver contacts’ evaporation. The corresponding layer sequences were ITO/PEDOT:PSS/PM6:Y7/PDINO/Ag for the Ref. (see Figure 1), ITO/CPE-Na/PEDOT:PSS/PM6:Y7/PDINO/Ag for the Bilayer devices, and ITO/CPE-Na-PEDOT:PSS/PM6:Y7/PDINO/Ag for the Mix. samples (Figure 3a). In the case of the Mix. cells, the best device performance due to an increase of fill factor (FF) was found for the mixing concentration ratio 1:4 between CPE-Na and PEDOT:PSS, as illustrated in the current density–voltage (*J–V*) curves of Figure S4a of the optimization. For the Bilayer devices, we deposited the CPE-Na layer at varying annealing temperatures, and the optimum performance was achieved at 90 °C, resulting in an increased fill factor (FF) and photocurrent, as illustrated in Figure S4b. We also measured dark *J*–*V* curves for different mixed ratios and for bilayers with different temperatures, showing similar behavior, as depicted in Figure S4c–d. Subsequently, the dark direct current (DC) mode resistance–voltage (*R*–*V*) curves were extracted from the dark *J–V* characteristics (see Section S2.1), and an increase in the shunt resistance (*R*_sh_) was observed in all the CPE-Na-based devices, as illustrated in Figure S4e–f. Corresponding to the *J*–*V* curve under dark conditions, the shunt resistance *R*_sh_ versus voltage has been extracted as illustrated in Figure S4e–f, and an increase in the shunt resistance *R*_sh_ was observed in all the CPE-Na-based devices.

### 3.2 Device Characterization

The initial performance of the studied devices with different HTLs demonstrates an improvement in FF and PCE for the two approaches of the mixed layer and bilayer devices compared to the reference sample that includes PEDOT:PSS as HTL. This is illustrated in the statistical analysis of Figure S5a–d and the *J–V* curves in Figure 3b corresponding to each structure’s “champion” cells, whose performance data are summarized in Table S4. The top-efficiency bilayer device exhibits a maximum PCE of 17.27%, with an open-circuit voltage (*V*_oc_) of 0.86 V, short-circuit current density (*J*_sc_) of 30.14 mA cm^–2^, and fill factor (FF) of 67%. The mixed layer (mix-CPE-Na) exhibits a noticeable increase in FF of 70% compared to the other devices in a sequence of high shunt resistance (*R*_sh_) of 496.2 Ω cm^2^. The device (mix-CPE-Na) exhibits a maximum PCE of 17.24, *V*_oc_ of 0.86 V, *J*_sc_ of 28.83 mA/cm^2^, and *R*_s_ of 1.38 Ω cm^2^. The reference device (ref-PEDOT:PSS) exhibits a maximum PCE of 16.52%, *V*_oc_ of 0.85, *J*_sc_ of 29.10 mA/cm^2^, FF of 0.67, *R*_s_ and *R*_sh_ of 1.35 and 264.6 Ω cm^2^, respectively. The inset images in Figure S5e display actual photographs of the bilayer CPE-Na, mix-CPE-Na, and ref-PEDOT:PSS devices for further illustration.

Optimization was taken to enhance the efficiency of the resulting NF-OPV devices by fine-tuning the annealing temperature of the bilayer devices, and the volume ratios present within the devices of the mixed layer. The current density–voltage (*J–V*) characteristics and comprehensive device performance parameters accompanied by statistical data for each optimization setup can be viewed in Figure S4 and Table S4.

The external quantum efficiency (EQE) shown in Figure 3c was tested for the devices bilayer-CPE-Na, mix-CPE-Na, and ref-PEDOT:PSS. The spectra resulted close to each other, which suggests that adding the CPE-Na within the devices does not affect the charge carrier transport properties of the PM6:Y7, provided the similar absorption evidenced in Figure 2b.^42^ In the right axis of Figure 3c, a slightly higher photocurrent generation is found for the Bilayer sample from the integral of the EQE spectra, and a photovoltaic bandgap of 1.39 eV is calculated for all models from the sigmoid fitting of the inflection point in the absorption threshold.^43^

The main charge carrier recombination mechanisms of the samples were studied via evaluation of the ideality factor (*m*), which is a parameter that indicates whether the recombination takes place by radiative or nonradiative mechanisms.^44^ The description of basic equations and formalisms for the ideality factor analyses, and the used measurement procedures, can be found in Section S2.1. Experimentally, the *J–V* curves were measured in the dark (see Figure 3d), and the *J*_sc_–*V*_oc_ curves were obtained under different illumination intensities (Figure 3e), using a solar simulator and a white light emitting diode, where the solar spectrum at standard AM1.5G, human eye photopic response (S), and white light emitting diode (LED) used in the impedance spectroscopy and ideality factor studies are shown in Figure S6. The resulting ideality factors are summarized in Figure 3f. In general, smaller values of *m* are shown for the Bilayer samples in comparison to those of the Ref. samples. This is in agreement with the FF increase and suggests that trap-mediated surface recombination has been reduced by including the CPE-Na interlayer between the ITO and the PEDOT:PSS. In particular, the dark *J–V* curves, where the concentration of photogenerated charge carrier is minimal, favoring the trap recombination,^45,46^ resulted in *m* values of 2.00, 1.95, and 1.70 for the Ref., Mix., and Bilayer samples, respectively. On the other hand, under illumination, not only did the photogeneration increase the band-to-band radiative recombination and thus reduce the ideality factors, but also the Mix. samples reported the lowest value of *m* = 1.22. The *m* behavior of the Mix. samples under illumination was also different from the Bilayer samples when comparing other techniques and conditions. This suggests that the device performance improvement by introduction of CPE-Na may be due to reduced trap recombination in the Bilayer strategy but should include other transport modifications when using Mix. layers.

Interestingly, the above-discussed values were obtained for relatively high voltage (↑*V*) conditions, i.e., within a forward bias range where the *J–V* or *J*_sc_–*V*_oc_ curves behave exponentially, without influence of series or shunt resistances, and corresponding to the range of operation under 1 sun illumination. However, under relatively lower voltages (↓*V*) and thus lower illumination intensities, the samples evidenced a different transport regime with *m* > 2. High ideality factors have been reported in several photovoltaic technologies.^47^ The most common explanations have been associated with energy disorder,^48^ parasitic interface series resistance in heterojunction devices,^49−53^ tunneling transport,^54−56^ multitrap recombination,^56−58^ and high grain boundary defect concentration.^59^

The saturation current density (*J*_sat_), which is applicable when the voltage is 0.17 V, as well as the maximum rate of exciton generation (*G*_max_), the generation rate (*G*_rat_), and the exciton dissociation probabilities (*P*_diss_) were calculated.^42,60^Figure S3c shows that the photocurrent increases linearly until *V*_eff_ = 0.17 V. Then mostly it becomes saturated for *V*_eff_ > 0.17 V for the three devices. This suggests an effective separation of charge carriers at low effective voltage. The results of these measurements and calculations from the *J*_ph_ versus *V*_eff_ curve are displayed in Table S5. We notice that involving CPE-Na as a buffer layer increases the charge carrier separations through the interface within the active layer. It exhibits *G*_max_ of 2.54 × 10^25^ m^–3^ s^–1^ and a generation rate *G*_rat_ of 2.47 × 10^27^ m^–3^ s^–1^, which is better than those for the reference device, where for ref-PEDOT:PSS, the *G*_max_ and *G*_rat_ are 2.47 × 10^25^ m^–3^ s^–1^ and 2.38 × 10^27^ m^–3^ s^–1^, respectively.

An increase in *J*_ph_ and *G*_max_ occurred by adding CPE-Na as a buffer layer, equating to a more efficient photocurrent response and a saturation state of saturation.^61^ Moreover, this is indicative of superb energy harvesting as a result of the efficient generation of excitons.^42,60^

*P*_diss_, which represents the probability of charge separation at the donor–acceptor interface, is notably 99.94% for the mix-CPE-Na device and 97.36% for the bilayer-CPE-Na device compared to the ref-PEDOT:PSS device, which is 96.6%. This suggests an efficient process of exciton dissociation at the tripling interface of the donor/acceptor, along with effective transport and collection of charge carriers between the photoactive layer and the electrode.^61^ This observation is in line with the highest fill factor (FF) for mix-CPE-Na as shown in Table S4

By means of impedance spectroscopy, we investigated the surface conductivity for the different HTLs, as presented in Figure S3d. In the Bode plots of Figure S3d, the constant conductivity plateaus toward low frequency indicate the steady state DC value. The results reveal an enhancement in DC conductivity with respect to the reference (CPE-Na-free) HTL by introducing the CPE-Na in bilayer and mixed approaches. This enhancement suggests that CPE-Na optimizes the electrical properties of the devices.^62^

**Figure 4:**
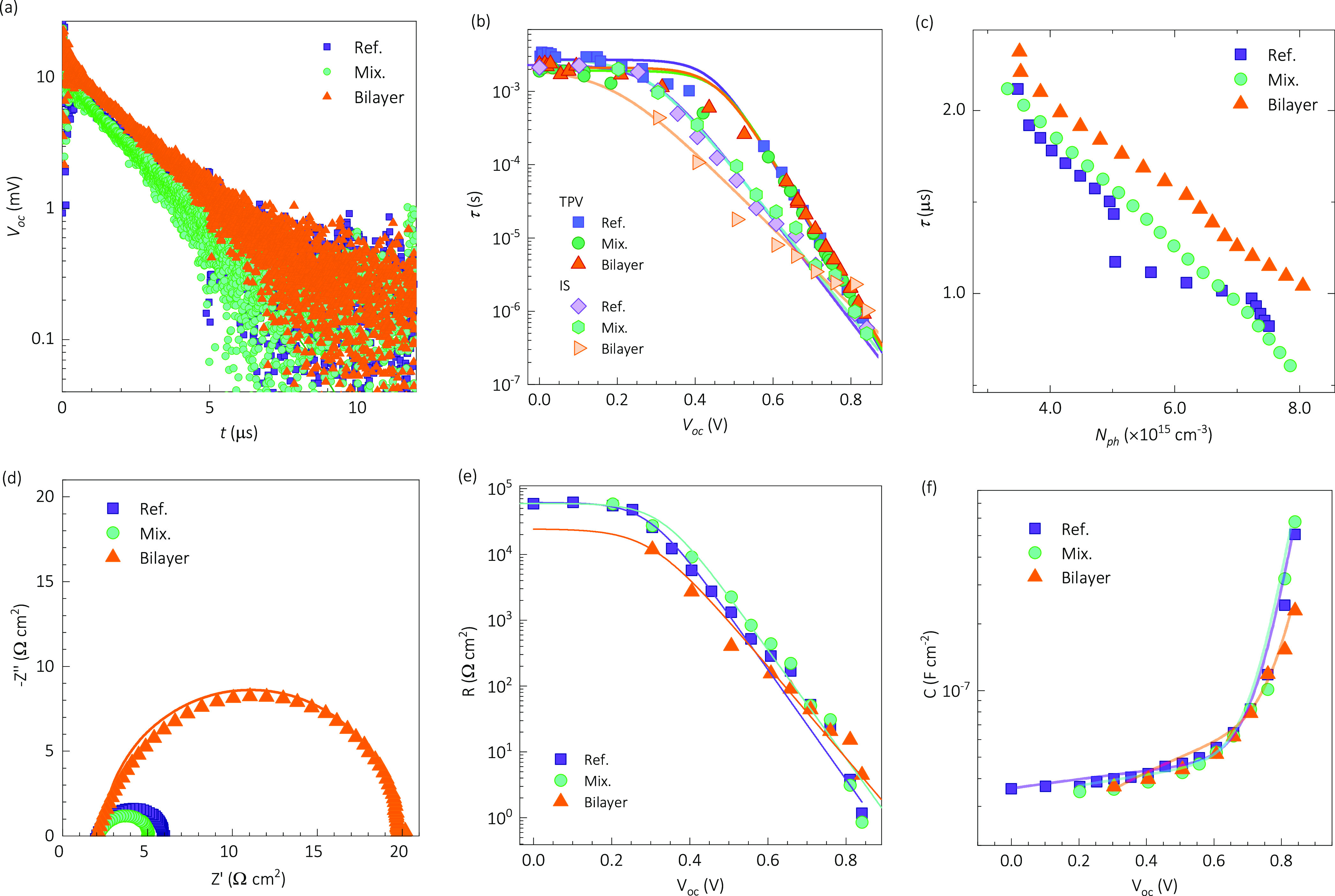
Transient and spectroscopic characterization: (a) photovoltage transients at a single illumination intensity; (b) characteristic response/recombination lifetime as a function of open-circuit voltage, and (c) photogenerated charge carriers for several illumination intensities; (d) illustrative impedance spectroscopy spectra in Nyquist plot representation at open-circuit for a single illumination intensity; and resultant (e) resistance and (f) capacitance from the equivalent circuit model fitting. See Section S2.3 for full data and further model details.

The changed behavior of the samples under different illumination intensities was also explored via transient (see Figure 4a–c) and spectroscopic (see Figure 4b,d–f) techniques. The fundamental and experimental details are summarized in Sections S2.3 and S2.4. Figure S7 shows the direct current mode equivalent circuit of a solar cell, while Figure S8 illustrates the theoretical background for the photocurrent density (*J*_ph_) versus the effective voltage (V_eff_), which are used to obtain the rate of exciton generation (*G*_max_), the generation rate (*G*_rat_), and the exciton dissociation probabilities (*P*_diss_).

The TPV measurements are shown in Figure S9, and a set of representative transients are shown in Figure 4a for a single illumination intensity with transparent monoexponential decays. The extracted recombination lifetimes (τ) from TPV are presented in Figure 4b as a function of the open-circuit voltage, showing similar behavior with a slight increase for the Bilayer sample over the others. The photoinduced charge extraction experiments were performed to assess the photogenerated density of charge carriers *N*_ph_ as a function of the DC illumination intensity, as shown in Figure S10. The resulting correspondence between recombination lifetime from TPV as a function of photogenerated charge carriers is presented in Figure 4c, where a clear difference is observed with higher lifetime values for the Bilayer sample compared to those in Ref. and the Mix. devices.

The IS technique was utilized in dark conditions, where the capacitance (*C*) was explored at 100 kHz changing the DC bias, and the equivalent circuit used for analytical modeling of impedance spectra is illustrated in Figure S11. The resulting Mott–Schottky plots in Figure S12 evidence clear p–i–n structure, i.e., a saturated plateau of the *C*^–2^–*V* curve toward reverse bias instead of a linear decrease starting – at least – from *V* = 0 V on. Accordingly, the traditional procedure cannot extract doping density nor built-in voltage.^11^ Instead, an exponential increase of the capacitance is observed in the *C*–*V* curves, which can be used to estimate a minimum doping density *N*_d,min_ value.^63^ Assuming no changes in dielectric constant and similar thicknesses, our estimations entail approximated values of *N*_d,min_*∼* 10^15^ cm^–3^, and the higher exponential increase of the CPE-Na-based samples suggests a *N*_d,min_ decrease in these HTLs (see Table S6).

Under illumination, the full IS spectra are shown in Figure S13. In contrast, Figure 4d shows a representative set of arcs in Nyquist representation for high-voltage/illumination conditions, where the recombination resistance is significantly larger for the Bilayer sample than those of the Ref. and Mix. devices. The spectra were fitted to the equivalent circuit model in Figure S11, and the resistance and capacitance results are in Figure 4e,f, respectively. The resistance behavior was parametrized to extract the ideality factors^44^ in Figure 3f, which agreed with the low voltage/illumination intensity condition results for the *J*_sc_–*V*_oc_ experiments.

Interestingly, the Bilayer sample presents the highest recombination resistance only for the highest illumination intensities/*V*_oc_ values, compared with the reference and Mix. samples. In the same range, the exponential increase of the capacitance is minimal for the Bilayer sample, possibly related to a lower density of states toward the bands.^64−66^

### 3.3 Stability Study

The stability of the fabricated devices was assessed via three different tests, designed to explore different aspects of the long-term evolution of the devices. These experiments included dark storage stability and operational and photostability tests under constant illumination.

#### 3.3.1 Dark Storage Stability

For the dark storage condition, the fabricated devices were placed in the glovebox under a nitrogen atmosphere in darkness after the initial and subsequent *J*–*V* curves (see workflowchart in Figure S14a). After each dark storage degradation cycle the *J*–*V* curve was measured under standard 1-sun illumination intensity, with the solar simulator. Besides the short time illumination stress during the *J*–*V* curves, the dark storage test explores the chemical inertness of the devices in the most simplistic approach where temperature, photon, and electron interaction are minimized.

**Figure 5:**
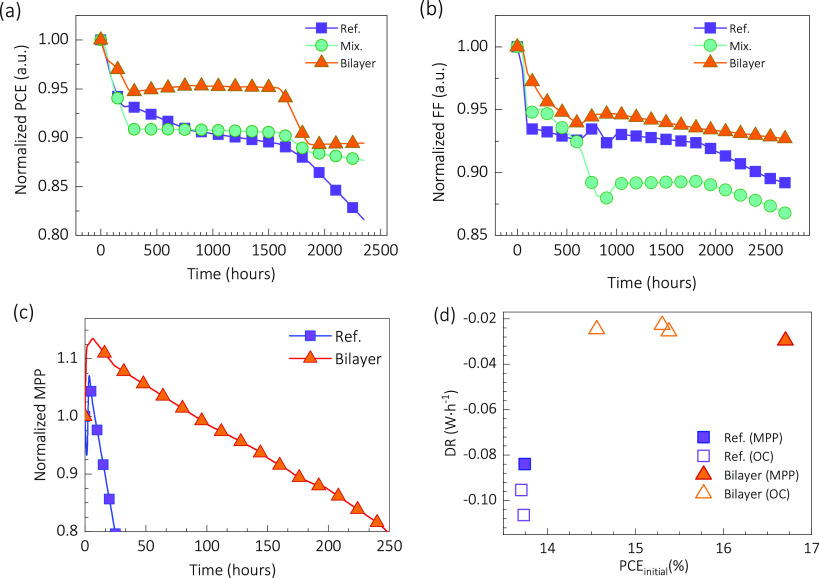
Device stability tests considering: (a,b) nitrogen dark storage and (c) operation under room conditions with MPP tracking under continuous 1 sun illumination (LED equivalent). The normalized PCE (a) and FF (b) for the dark storage test correspond to systematic *J–V* curves measured with standard 1 sun illumination (solar simulator). The degradation rates versus initial power conversion efficiencies in (d) include data from both the dark storage and the illuminated stability tests.

The results of the storage stability test are presented in Figure 5a,b in terms of the normalized PCE and FF, which in both cases indicate lower degradation for the bilayer sample. Figure 15Sa shows *J*_sc_ versus time where *J*_sc_ remained nearly constant, reaching 95% of the initial value (t95) after 2500 h for bilayer-CPE-Na. For mix-CPE-Na, it remained above t95. Moreover, as seen in Figure 15Sb, the *V*_oc_ stayed almost constant throughout the test until 2500 h. In summary, the degradation test in dark storage conditions shows a significant impact for the introduction of CPE-Na into the HTL with the PEDOT:PSS.

#### 3.3.2 Operational Stability (MPP) Under Constant Illumination

The second stability test simulated the operational conditions by setting the sample in a nonisolated holder with maximum power point (MPP) tracking under continuous 1 sun equivalent white LED illumination. The temperature of the degradation chamber was 46 °C ± 3 °C. The MPP tracking consists of applying the voltage corresponding to the MPP with systematic corrections accounting for the stability of the sample (see workflowchart in Figure S14b), and the results in normalized percentages are shown in Figure 5c. This stability test simulates the operational conditions of solar cells, where illumination, temperature, atmosphere, and voltage biasing determine the electrical response of the sample.

The use of sole PEDOT:PSS as the HTL exhibits a more rapid decrease, losing 50% of its initial power within 72 h. On the other hand, involving CPE-Na within the device shows much better stability: the bilayer approach, including both CPE-Na and PEDOT:PSS in the HTL, lost 20% of its initial power over a much more extended period of 275 h. This significantly slower MPP degradation rate suggests that adding CPE-Na benefits the device’s stability.

#### 3.3.3 Photo Stability (Open Circuit) Under Constant Illumination

Unlike the MPP tracking under illumination, where all the operational parameters are present, one can focus on the photostability by setting the device in open-circuit conditions where no current is flowing. In this state, over heating due to current dissipation and any other degradation mechanism associated with external voltage bias is minimized. Moreover, since the employed homemade degradation chamber was only able to perform the MPP tracking to one cell at the time, during each operational stability test, the remaining disconnected pixels/diodes in the substrate are subjects of a light stability test in open-circuit condition (see workflowchart in Figure S14c).

The presence of CPE-Na could protect against the thermal and photonic stresses applied during testing, contributing to an extended device lifetime. Given its chemical properties, CPE-Na may help to improve moisture resistance, enhance morphological stability, and ensure better interfacial compatibility, resulting in a more durable and efficient device.

We have measured the pH values for both PEDOT:PSS and the mixed solution of PEDOT:PSS and CPE-Na, and the results indicate a pH of 2.06 for the PEDOT:PSS solution and 2.45 for the mixed solution (PEDOT:PSS/CPE-Na). The acidity of PEDOT:PSS is thereby affected by the addition of a CPE-Na solution, leading to a slightly higher pH. We hypothesize that the increase in pH may contribute to the improved stability of the OSCs. A more neutral environment, compared to the inherently acidic PEDOT:PSS, could reduce the degradation of active materials and interfaces in the device, leading to enhanced long-term stability

The stability test results can also be analyzed using degradation rates (DRs). In a dynamic approach, a time-dependent or in situ degradation rate can be defined as the time derivative DR = d*X*/d*t* of the performance parameter *X*; e.g., *X* = PCE. However, this definition results in comparable information to the time evolution of the parameter *X*, which hinders the comparison between samples, and even numerical artifacts can arise due to the uneven behavior of the experimental data. On the other hand, an overall stability test degradation rate has been proposed^3^ as
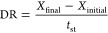
1which quantifies the change of the parameter *X*; e.g., PCE or MPP, between the initial and final states during the stability test of duration *t*_st_. Figure 5d shows the degradation rate versus the initial efficiency values (PCE_initial_), where *t*_st_ is taken as the t90 for the dark storage and t80 for the operational test. In addition, the data corresponding to the light stability test are also included. The higher absolute value degradation rates (more negative DR values) are evidenced for the reference sample compared with the Bilayer cells. Interestingly, when comparing the MPP and OC light tests, the DR values are nearly identical for the Bilayer samples and slightly larger at OC for the reference cells. This may indicate that CPE-Na hinders reactive pathways triggered during OC when no current flows through the cell.

## 4 Conclusions

In summary, this study demonstrates the beneficial impact of utilizing the conjugated polyelectrolyte PCPDTPhSO3-Na (CPE-Na) along with PEDOT:PSS, within the hole transport layer in nonfullerene organic solar cells comprising PM6:Y7 as the active layer and PDINO as the electron transport material. Two approaches were tested, the bilayer and mixture methodologies, and not only did the performance of the CPE-Na-based samples decrease, but several improvements were also observed in charge extraction and reduced nonradiative charge carrier recombination. Additionally, the introduction of CPE-Na was also found to increase the dark storage and operational stability. Remarkably, between the two methodologies, the bilayer approach resulted in the best performance over the mixed layer alternative. This suggests that the interface between the PEDOT:PSS and the ITO is not only a source of recombination centers that require optimization but also a significant contributing factor to the reactivity and subsequent instability of the electrical response of the device.

## Supporting Information Available

Devices fabrication for the optimization process and characterization; the energy diagram data; morphological characterization (FESEM, AFM, and contact angle); PL spectra, *J*_ph_ versus *V*_eff_ accompanied by the resulting parameters of this measurement, surface conductivity; *J*–*V* curve (light and dark) for the optimized devices; statistical analysis of *J*–*V* curve parameters accompanied by the performance parameters; photon flux of the used solar spectrum; the analytical models that are used in data analysis, TPV and PL-CE data and figures; Mott–Schottky representation; fitted parameters for the EC simulation of IS spectra; degradation test work flows and the results (PDF)
