# Supplementary material for: CPE-Na-Based Hole Transport Layers for Improving the Stability in Nonfullerene Organic Solar Cells: A Comprehensive Study
Source: ACS Appl Mater Interfaces. 2024 Mar 25;16(13):16317–27. doi: 10.1021/acsami.4c01154 (PMC10995908; doi:10.1021/acsami.4c01154)
Supplement: Supplementary file 1 — am4c01154_si_001.pdf [file am4c01154_si_001.pdf]

Supporting information:

CPE-Na-based Hole Transport Layers for improving the stability in Non-Fullerene Organic Solar Cells: A Comprehensive Study

Mohamed Samir[1‡], Enas Moustafa[1,3‡], Osbel Almora[1], Magaly Ramírez-Como[4], Maria Pilar Montero-Rama[1], José G. Sánchez[2], Emilio Palomares[2,5], Josep Pallarès[1], Lluis F. Marsal[1*]

[1]Department of Electronic, Electric and Automatic Engineering, Universitat Rovira i Virgili, Tarragona, 43007, Spain

[2]Institute of Chemical Research of Catalonia-CERCA (ICIQ-CERCA), Tarragona, 43007, Spain.

[3]Science and Engineering of Renewable Energy Department, Faculty of Postgraduate Studies for Advanced Science, Beni Suef University, Beni Suef, 62521, Egypt.

[4]Sección de Estudios de Posgrado e Investigación, UPIITA Instituto Politécnico Nacional, Mexico City, 07340, Mexico.

[5] Institución Catalana de Investigación y Estudios Avanzados (ICREA) Barcelona, 08010, Spain.

*lluis.marsal@urv.cat

S1. Fabrication and Material Characterization

Optimization of the fabricated Devices

In the bilayer devices, we used different annealing temperatures for CPE-Na layer, 50C, 90C and 120C. The best performance was provided by annealing temperature of 90C/10min as shown in Figure S1 for the J-V curve under 1 sun illumination and dark illumination. Based on that, we choose 90C/10min to complete the characterization.

For the mixed layer devices, we prepared three volume ratios to optimize the best ratio of the prepared mixture. Ratio (4:1) of PEDOT:PSS to CPE-Na shows better PCE than ratio (2:1) and (8:1) as shown in Figure S1.

Table S1: Data used for the energy diagram of Figure 1 (main manuscript)

Material	Work function (eV)	Bandgap (eV)
Indium tin oxide (ITO)	4.15-4.3[1]; 4.4-4.5[2]; 4.5-4.75[3]	-
Poly(3,4-ethylenedioxythiophene) polystyrene sulfonate (PEDOT:PSS)	-	3[4]
$(C_{68}H_{76}F_2O_2S_8)_n$, PBDB-T-2F (PM6)	-	1.9 [5]
$C_{82}H_{86}Cl_4N_8O_2S_5$, BTP-4Cl (Y7)	-	1.6[4]
$C_{34}H_{32}N_4O_6$ (PDINO)	-	2.5[4]
Ag	4.14-4.46;[6] 4.26[7]; 4.35[8]	-

(a)
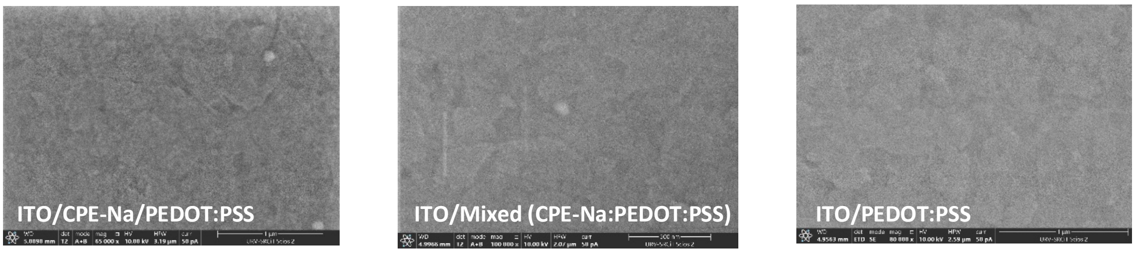

(b)
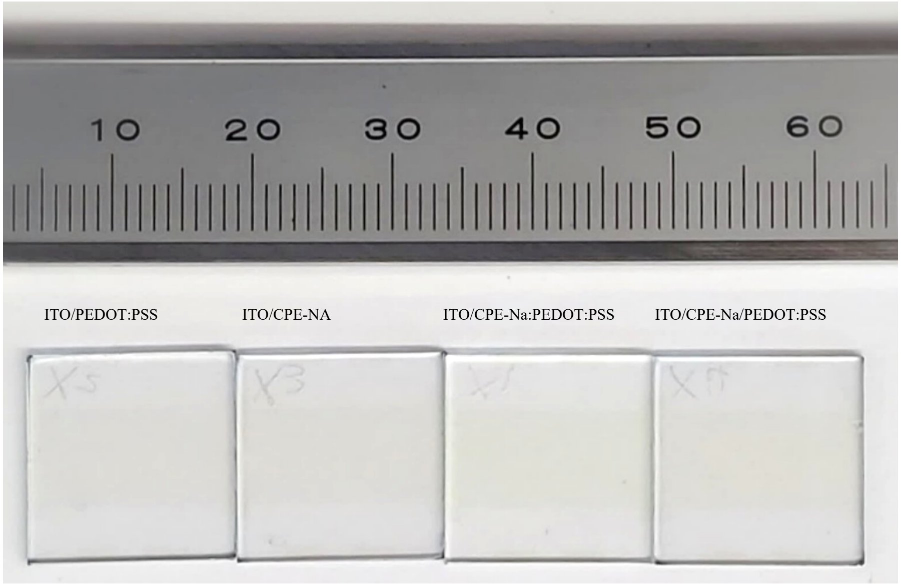

Figure S1. Morphological characterization of HTL approaches (a) scanning electron microscopy, and (b) photography images for several sequence of layers, as indicated.

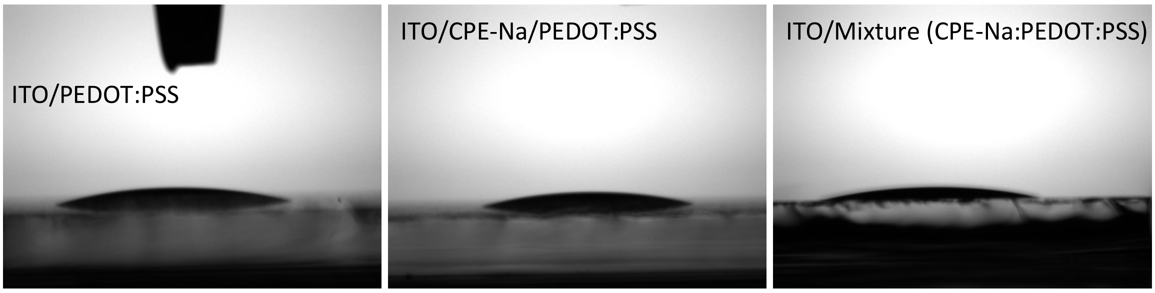

Figure S2. Contact angle for a drop of PM6:Y7 on the prepared layers. The mean angles are summarized in **Table S2**.

Table S2: Summary of the results from the S3 in **Figure S2**

**Sample**	**Mean angle (degrees)**
ITO/PEDOT:PSS	16.7 ±0.3
ITO/CPE-Na/PEDOT:PSS	14.2 ±0.2
ITO/Mixture (CPE-Na:PEDOT:PSS)	13.3 ±0.4

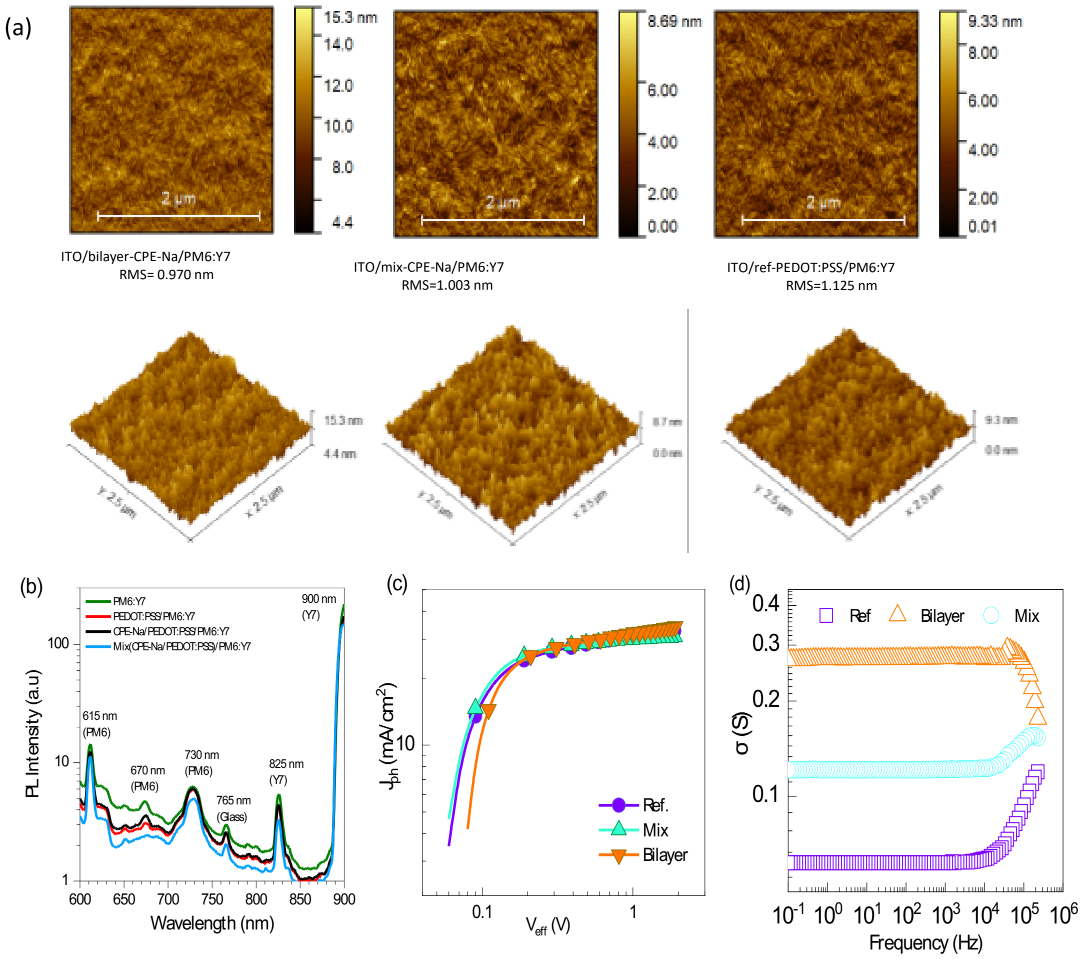

Figure S3. (a) AFM topography and 3D images of the PM6:Y7 active layer on the fabricated layers, (b) photoluminescence (PL) spectra of several sequences of layers, as indicated in each case. (c) J_{ph} versus V_{eff} (d) Surface conductivity versus frequency for the different HTL layers, as indicated. The distance between silver electrodes was of 1 mm.

Table 3S: Summary of the PL peaks in **Figure S3b**

Peaks (nm)	Molecule	Reference
615	PM6	[9]
670	PM6	[10]
695	PM6	[11]
730	PM6	[12]
765	Glass	[13]
825	Y7	[14]
900	Y7	[11]

S2. Device characterization

S2.0. Experimental device performance

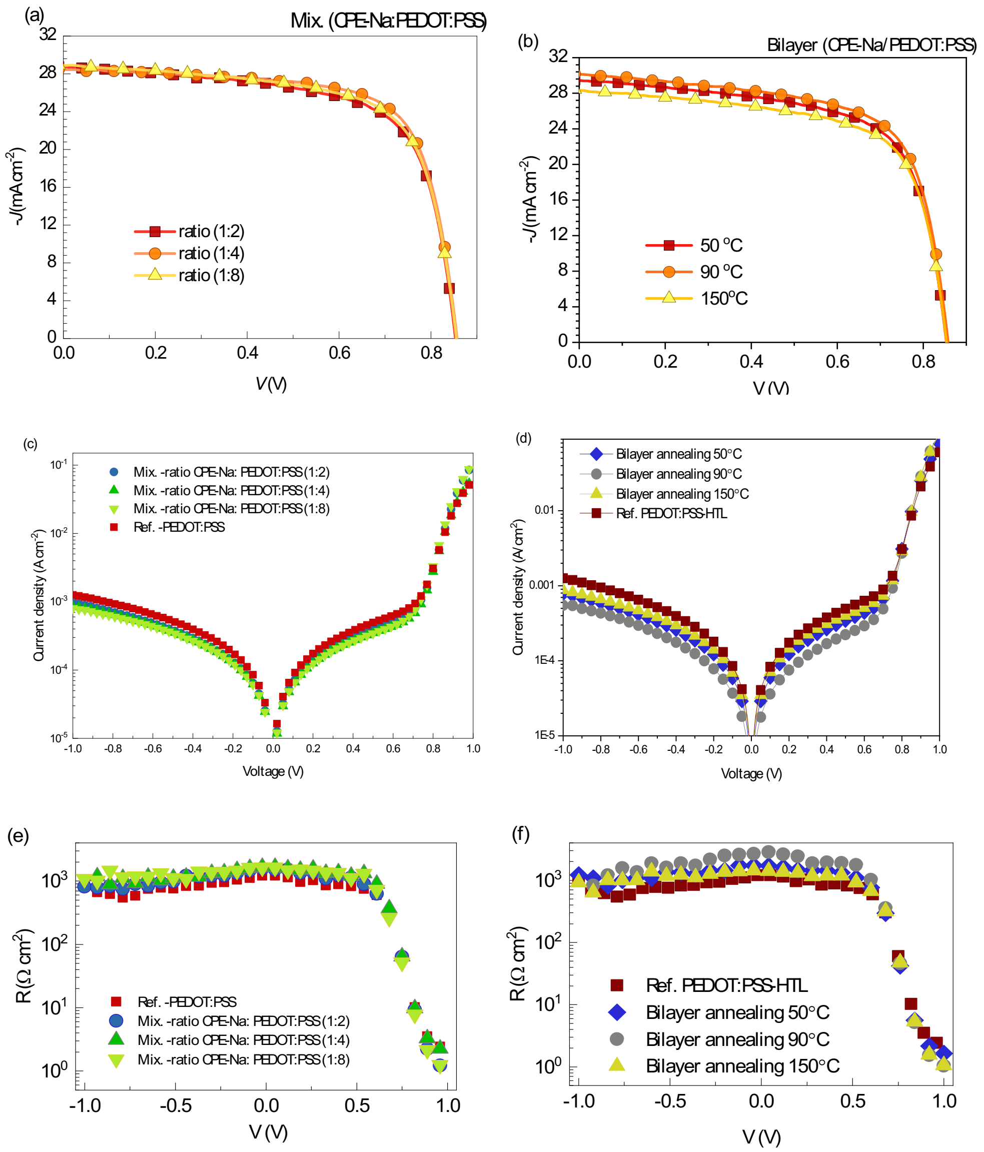

Figure S4. Current-voltage characterization under (a, b) solar simulator and (c-f) in dark for (a, c, e) mixture and (b, d, f) bilayer samples with different blend ratios and annealing temperature during 10 minutes, respectively. The dark DC resistances in (e) and (f) correspond to (c) and (d), respectively.

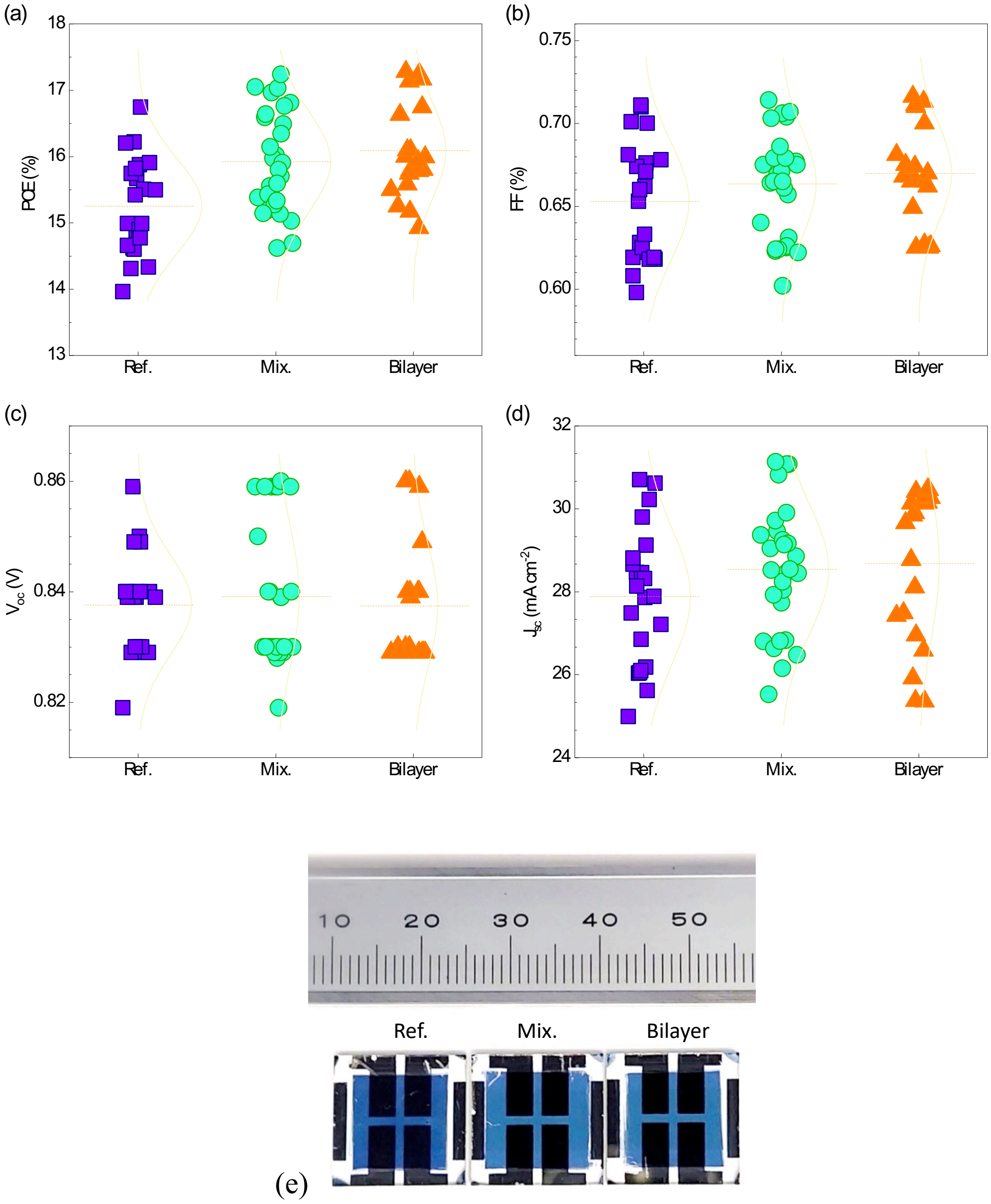

Figure S5. Statistical analysis of current-voltage characterization under solar simulator in terms of the performance parameters: (a) power conversion efficiency, (b) fill factor, (c) open-circuit voltage and (d) short-circuit current density. In (e) a photography of the representative samples indicates the sample size and physical appearance.

Table S4: Performance parameters for the maximum efficiency devices in Figure 3b (in the main manuscript) measured under solar simulator illumination.

**Device**	**V_{oc}** (V)	**FF** (%)	**J_{sc}** (mA cm^{-2})	**PCE** (%)	**PCE** (max)	**R_s** ($\Omega.cm^2$)	**R_{sh}** ($\Omega.cm^2$)
bilayer-CPE-Na (90 C)	0.86 ± 0.01	67 ± 0.1	30.14 ± 0.01	17.22 ± 0.05	17.27	1.31 ± 0.01	261.5 ± 2.44
mix-CPE-Na ratio (1:4)	0.86 ± 0.01	70 ± 2	28.83 ± 0.73	17.07 ± 0.13	17.24	1.38 ± 0.08	496.2 ± 195
ref-PEDOT:PSS	0.85 ± 0.01	67 ± 1	29.10 ± 0.14	16.52 ± 0.33	16.74	1.35 ± 0.01	264.6 ± 14.7
bilayer-CPE-Na 50 C	0.85 ± 0.00	66 ± 1	29.45 ± 0.01	16.60 ± 0.01	16.62	1.32 ± 0.01	272.7 ± 26.6
bilayer-CPE-Na 150C	0.85 ± 0.01	67 ± 1	28.22 ± 0.07	16.11 ± 0.05	15.99	1.26 ± 0.01	248.8 ± 20.9
mix-CPE-Na ratio (1:2)	0.85 ± 0.01	67 ± 0.1	28.74 ± 0.07	16.46 ± 0.04	16.52	1.20 ± 0.01	305.1 ± 49.0
mix-CPE-Na ratio (1:8)	0.86 ± 0.01	067 ± 1	28.96 ± 0.14	16.70 ± 0.16	16.81	1.32 ± 0.02	299.9 ± 14.8

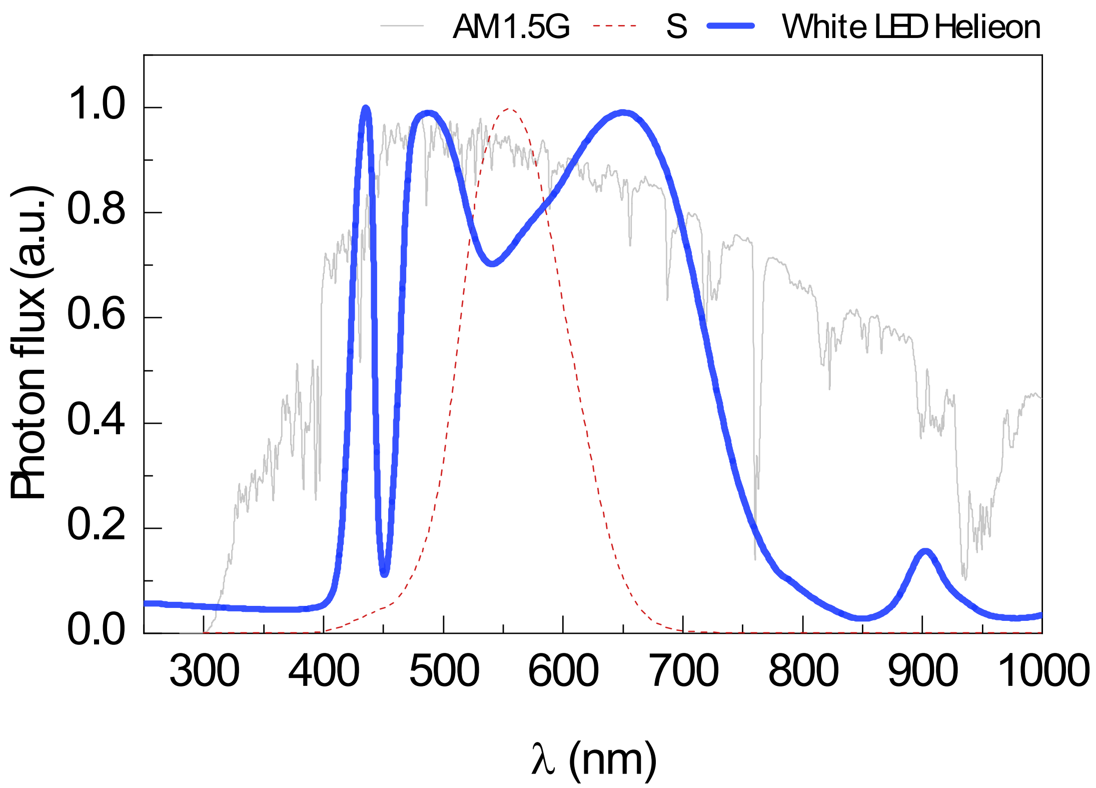

Figure S6. Standard AM1.5G solar spectrum, human eye photopic response (S), and white light emitting diode (LED) used in the impedance spectroscopy and ideality factor studies. The assistance of Dr. Laura K. Acosta for the measurement of the LED spectrum with Fluorolog Horiba Jobin Yvon spectrofluorometer is acknowledged.

S2.1. Analytical models for the current-voltage characteristics and derived parameters

The basic direct current (DC) mode equivalent circuit (EC) of a solar cells combines the ideal diode behavior, with the parasitic effects of series ohmic voltage drops (V_{Ω}) through the series resistance (R_s), leakage recombination current shunt resistance (R_{sh}) and the photocurrent generation as a current source. All of this is illustrated in the schemed EC in **Figure S7**, where the current density J through the diode is the empirical form of Shockley's equation[15-16]

$$J = J_s \left(\exp\left[\frac{qV}{m\, k_B T}\right] - 1 \right) \tag{S1}$$

where J_s is the dark reverse bias saturation current density, q is the elementary charge, k_B is the Boltzmann constant, T is the temperature, V is the voltage at the diode, and m is the ideality factor. For an ideal p-n homojunction diode in the dark, m approaches 2 when trap-mediated Shockley-Read-Hall (SRH)[17-18] non-radiative recombination is the main transport mechanism and m approaches 1 when diffusion current dominates.[19] In more realistic devices, and including the photocurrent (J_{ph}) generation of charge carriers, the current can be deduced from the EC in **Figure S7** by applying Kirchhoff's circuit laws, resulting

$$J = J_s \left(\exp\left[\frac{q(V - R_s J)}{m\, k_B T}\right] - 1 \right) + \frac{(V - R_s J)}{R_{sh}} - J_{ph} \tag{S2}$$

The transcendent Equation (S2) does not have analytic solution, but it can be either utilized with numerical methods or reduced to simpler particular cases. Importantly, differently to its physical meaning in Equation (S1), the voltage V in Equation (S2) is the external voltage applied by the power source or external load.

Under illumination conditions, the ideality factor can also approach $m \approx 1$ when radiative band-to-band recombination is the main transport mechanism[20] or result in high values $m > 2$ due to several processes,[21] including energy disorder,[22] parasitic interface series resistance in heterojunction devices,[23-27] tunneling transport,[28-30] multi-trap recombination,[30-32] high grain boundary defect concentration.[33]

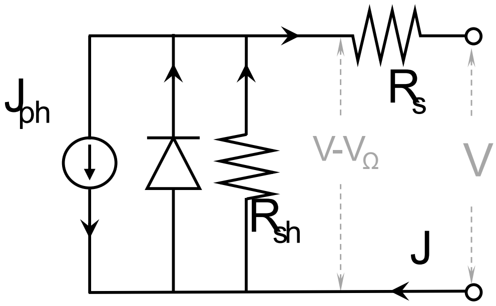

Figure S7. Direct current mode equivalent circuit of a solar cell. Here $V_\Omega = R_s J$. Reproduced with permission from ref.,[34] licenced under a Creative Commons Attribution - NonCommercial - NoDerivatives 4.0 International (CC BY-NC-ND 4.0) licence.

More than 20 experimental methods have been suggested for determining the ideality factor.[35-38] In the dark, the $J - V$ curve ($J_{ph} = 0$) of the device is measured and an intermediate forward bias voltage section of the curve can be fitted to the expression

$$J \approx J_s \exp\left[\frac{qV}{m\, k_B T}\right] \tag{S3}$$

which can be obtained from Equation (S2) by neglecting (i) ohmic series resistance losses ($V \gg R_s J$); (ii) thermal generation ($\exp[qV/m\, k_B T] \gg 1$); and (iii) leakage currents ($J_s \exp[qV/m\, k_B T] \gg V/R_{sh}$).

Under different illumination intensities, the device can be set to open-circuit (OC, $J = 0$ A·cm^{-2}), and Equation (S2) can be modified as

$$J_{ph} = J_s \left(\exp\left[\frac{qV_{oc}}{m\, k_B T}\right] - 1\right) + \frac{V_{oc}}{R_{sh}} \tag{S4}$$

Similarly, the short-circuit condition (SC, $V = 0$ V) can be set and the McLaurin series can be used to write Equation (S2) in the form

$$J_{sc}\left(1 + \frac{R_s}{R_{sh}} + \frac{qR_s J_s}{m\, k_B T}\right) = -J_{ph} \tag{S5}$$

From Equation (S5), no DC bias dependency is expected and a voltage-independent proportionality $J_{sc} \propto J_{ph}$ is safe to be assumed. Moreover, assuming $R_s \ll R_{sh}$ and room temperature ($R_s J_s \ll m\, k_B T/q$), the absolute valued of the short-circuit and photo currents can be fairly approached $J_{sc} \cong J_{ph}$. Therefore, the relation between the absolute values of J_{sc} and V_{oc} can be taken as

$$J_{sc} \cong J_s\left(\exp\left[\frac{qV_{oc}}{m\,k_BT}\right]-1\right)+\frac{V_{oc}}{R_{sh}} \tag{S6}$$

Similarly, in the proper voltage range, where one can neglect thermal generation ($\exp[qV_{oc}/m\,k_BT] \gg 1$) and leakage currents ($J_s\exp[qV/m\,k_BT] \gg V_{oc}/R_{sh}$), resulting in

$$J_{sc} \cong J_s\exp\left[\frac{qV_{oc}}{m\,k_BT}\right] \tag{S7}$$

In terms of resistance, its differential definition is given as[39-41]

$$R=\left(\frac{\mathrm{d}J}{\mathrm{d}V}\right)^{-1} \tag{S8}$$

The definition of Equation (S8) can be numerically applied to any experimental $J-V$ data, but also in an analytical form on the empirical Shockley's equation in the dark, resulting in

$$R=R_{th}\exp\left[-\frac{qV}{m\,k_BT}\right] \tag{S9}$$

where is the thermal recombination resistance is given as

$$R_{th}=\frac{m\,k_BT}{qJ_s} \tag{S10}$$

Moreover, with a more realistic approach, definition (S8) can be applied to the Equation (S2) under the assumption of negligible ohmic series resistance voltage losses ($V \gg R_sJ$), resulting in

$$R=\frac{R_{sh}}{1+\exp\left[\frac{q(V-V_{sh})}{m\,k_BT}\right]} \tag{S11}$$

where the leakage-current voltage threshold is not only the voltage by which the resistance is $R_{sh}/2$, but also it is defined as

$$V_{sh}=\frac{m\,k_BT}{q}\mathrm{Ln}\frac{R_{th}}{R_{sh}} \tag{S12}$$

Notably, since $R_{th} > R_{sh}$, V_{sh} will be a multiple of the thermal voltage and the smaller the R_{sh} the higher the V_{sh} and the lower the fill factor of the cell under illumination. Furthermore, the ideality factor can also be extracted from the resistance by using Equation (S11).

Up to this point, the ideality factor was considered as a constant parameter characterizing a transport regime. However, in practice, the transition between different regimes is not always clear and a more dynamic definition should be adopted. For instance, Equation 2 can be reordered to obtain

$$m = \frac{q(V - R_s J)}{k_B T}\left(\mathrm{Ln}\left[\frac{J\left(1 + \frac{R_s}{R_{sh}}\right) + J_{sc} + J_s - \frac{V}{R_{sh}}}{J_s}\right]\right)^{-1} \tag{S13}$$

Which can be simplified to

$$m = \frac{qV}{k_B T}\left(\mathrm{Ln}\left[\frac{J + J_{sc}}{J_s}\right]\right)^{-1} \tag{S14}$$

Another interesting property arises if the logarithm term of equations (S13) and (S14) is cleared and both members are derived with respect to the voltage. Then,

$$m = \gamma \frac{q}{k_B T}\frac{J}{\left(\frac{\mathrm{d}J}{\mathrm{d}V}\right)} = \gamma \frac{q}{k_B T} J\, R \tag{S15}$$

Where the resistance is that of Equation (S8) and the parasitic resistance coefficient is given by

$$\gamma = \left(1 - R_s \frac{\mathrm{d}J}{\mathrm{d}V}\right)\frac{\left(1 + \frac{R_s}{R_{sh}} + \frac{(J_s + J_{sc})}{J} - \frac{V}{R_{sh}\, J}\right)}{\left(1 + \frac{R_s}{R_{sh}} - \frac{1}{R_{sh}\frac{\mathrm{d}J}{\mathrm{d}V}}\right)} \tag{S16}$$

Note that, in dark and for $R_s \ll R_{sh}$, it is not difficult to find a convenient voltage range where $V \ll R_{sh}J$, $R_s \ll R \ll R_{sh}$ and $J_s \ll J$, reducing $\gamma \cong 1$.

S2.2. Theoretical background for the photocurrent density (J_{ph}) versus the effective voltage (V_{eff})

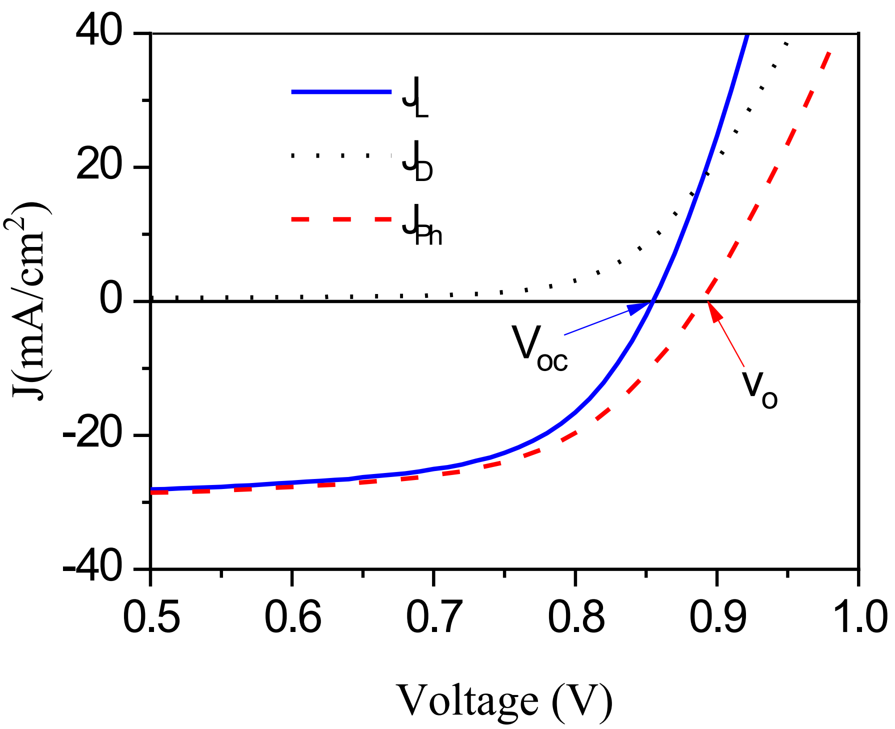

Figure S8: J_{ph}, J_L and J_D as a function of voltage

The dependence of the photocurrent density (J_{ph}) on the effective voltage (V_{eff}), recorded under illumination at 100 mW.cm^{-2}

$$J_{ph} = J_{light} - J_{dark} \quad [42] \qquad \text{(S17)}$$

where J_{light} and J_{dark} are the current densities under illumination and in dark, respectively.

$$V_{eff} = V_o - V \quad [42] \qquad \text{(S18)}$$

Where V_o refers to the voltage at which the photocurrent density (J_{ph}) is zero, meaning the rate of light-generated current (J_{light}) equals the rate of dark current (J_{dark}), and V is the designated applied voltage. Evidently, J_{ph} exhibits a linear growth with the voltage at a low effective voltage (V_{eff}), and subsequently plateaus when V_{eff} reaches a high enough value [42]

$$P_{diss} = \frac{J_{sc}}{J_{sat}} \quad [4] \qquad \text{(S19)}$$

where P_{diss} is the exciton dissociation probabilities.

Assuming that every photogenerated exciton dissociates and contributes to the current when the cell operates in the saturation regime (due to the sufficiently high electric field), we were able to determine the values for the maximum exciton generation rate (G_{max}) using the next equation

$$G_{max} = \frac{J_{sat}}{q\,L} \quad (S20)$$

[4]

where G_{max} is the maximum photon-to-charge carrier generation rate q is the electronic charge and L is the thickness of the active layer.

$$G_{rat} = G_{max} P_{diss} \quad (S21)$$

[4]

where G_{rat} is the generation rate of the free charge carriers.

Table S5: Summary of the resulting parameters from J_{ph} versus V_{eff} characteristic

**Device**	**J_{sc} (mA/cm2)**	**J_{sat} (mA/cm2)**	**P_{diss} %**	**G_{max} $\times10^{25}$ m^{-3}. s^{-1}**	**G_{rat} $\times10^{27}$ m^{-3}. s^{-1}**
bilayer-CPE-Na	30.14	30.96	97.36	2.54	2.47
mix-CPE-Na	28.83	28.84	99.94	2.36	2.33
Ref-PEDOT:PSS	29.10	30.12	96.60	2.47	2.38

S2.3. Photoinduced transient methods: TPV and PICE

S2.3.1. TPV and PI-CE fundamentals[43]

In the transient photovoltage (TPV) technique, a small perturbation light pulse is applied to the sample on top of a steady state illumination, also known as direct current (DC) mode background illumination. The sample is set at open circuit, so the DC photovoltage $V_{oc,DC}$ experiences an abrupt increase ΔV_{oc} and an exponential decay with the characteristic recombination lifetime τ. The photovoltage increase is expected with values no larger than the thermal voltage (ΔV_{oc} ≈26 mV at room temperature) and the subsequent decay can be fitted to the mono-exponential function

$$V_{oc} = V_{oc,DC} + \Delta V_{oc} \exp\left[-\frac{t}{\tau}\right] \quad \text{(S22)}$$

By changing the DC illumination intensity and adjusting the perturbation, a series of lifetime values τ as a function of the DC photovoltage $V_{oc,DC}$ can be obtained.

In the photoinduced change extraction (PICE) technique, the sample is first kept at open-circuit under a DC illumination intensity during a charging time. During this first step the dielectric, also known as geometrical, and chemical capacitances of the sample accumulate change carriers at the electrodes and the energy bands, respectively. Subsequently, the DC illumination is simultaneously switched off at the same time the sample is short-circuited. Then the capacitor (the sample) discharge current at short circuit in the dark is measured and integrated to account for the extracted charge. The process is repeated over different DC illumination intensities so the behaviour of the extracted charge over the photovoltage can be obtained. Since the relevant information to be acquired is that of the charge carrier density stored in the energy band via the chemical capacitance C_μ, the dielectric capacitance-related charge $Q_g = C_g V_{oc}$ should be subtracted from the total integrated charge.

S2.3.2. TPV and PI-CE data and parameterization

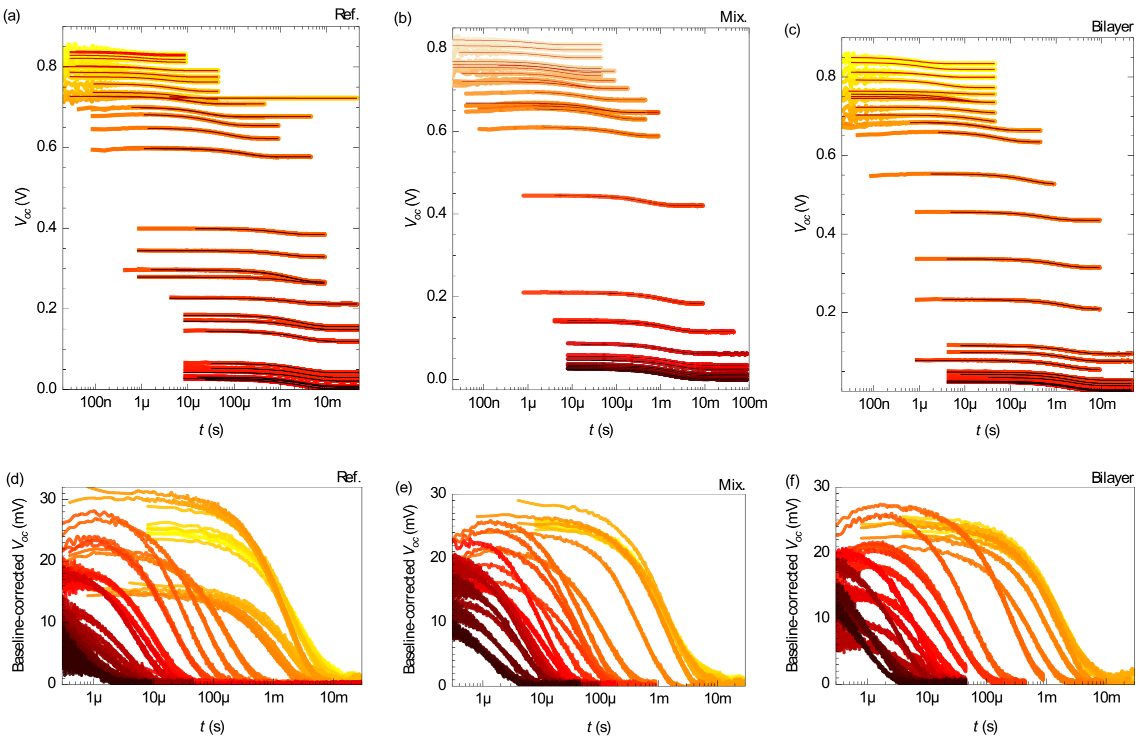

Figure S9. Transient photovoltage (TPV) measurements for three representative samples, as indicated. The top panel (a-c) presents the raw signal and the bottom panel (d-f) shows the transients corrected with respect to the DC photovoltage.

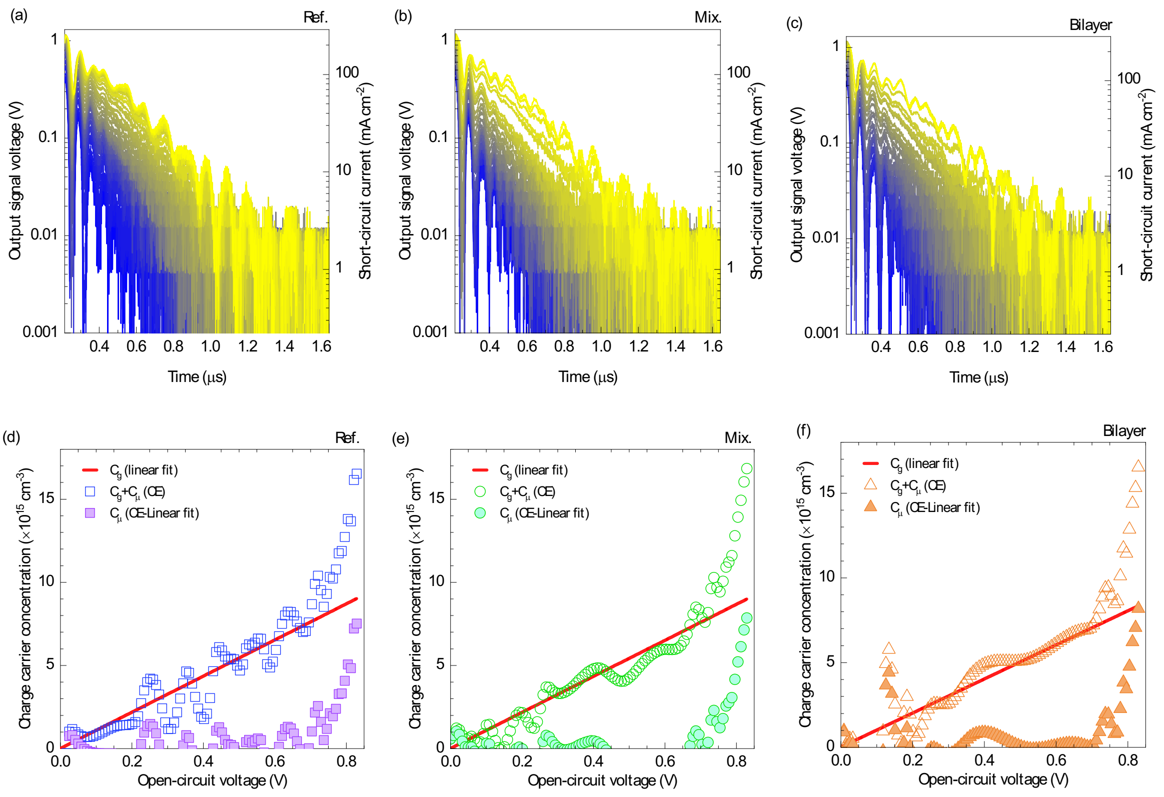

Figure S10. Photoinduced charge extraction (CE) for the three representative samples studied by TPV (see **Figure S9**). (a-c) shows the output signal voltage and the short circuit current versus the pulsed time while (d-f) shows the extracted charge carrier concentration against open-circuit voltage for the Ref., Mix. and Bilayer devices, as indicated. In (a-c), the lighter the colour the higher the illumination intensity and thus the photovoltage. In (d-f), the geometrical capacitance is separated from the chemical contribution, as indicated (see also Section S2.3.1)

S2.4. Impedance characterization

S2.4.1 IS fundamentals

The impedance spectroscopy was measured with an Impedance analyzer (HP 4192A LF). The samples were illuminated with a white LED (see **Figure S6**) at different steady-state illumination intensities, then the open-circuit voltage (V_{oc}) was stabilized before applying the corresponding forward bias that cancels the direct current (DC) operation: the so-called quasi-open-circuit condition. Upon these DC conditions the 15 mV perturbation was applied for measuring the impedance spectroscopy (IS) as a function of the V_{oc} for each illumination intensity. Subsequently, the IS spectra were fitted to the equivalent circuit (EC) model of Figure S11.

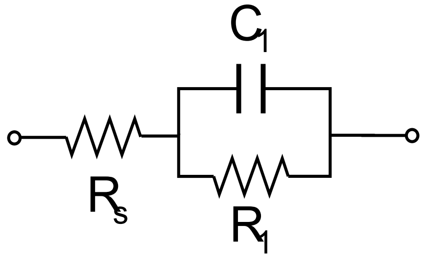

Figure S11. Equivalent circuit used for analytical modelling of impedance spectra and subsequent numerical fitting. Here R_s is a series resistor, R_1 stands as a recombination resistor, and C_1 is a capacitor.

The resistance values extracted from the EC model were subsequently parameterized as a function of V_{oc}, following the equation

$$R = R_{s0}\left(1+\frac{V_{oc}}{V_b}\right)+\frac{R_{sh}}{1+\exp\left[\frac{q(V_{oc}-V_{sh})}{m\,k_B\,T}\right]} \qquad \text{(S23)}$$

where R_{s0} is the series resistance; R_{sh}, the shunt resistance; m, the ideality factor; k_B, the Boltzmann constant; q, the elementary charge; T, the temperature; V_b is an effective series contact barrier voltage, and the equilibrium recombination voltage is a function of the dark reverse bias saturation current J_s as[41] $V_{sh} = mk_BTq^{-1}\mathrm{Ln}[q\,J_s\,R_{sh}(mk_BT)^{-1}]$.

Similarly, the capacitance values extracted from the EC model were subsequently parameterized as a function of V_{oc}, following the equation

$$C = C_d\left(1+\frac{V_{oc}}{V_d}\right)+C_n\exp\left[\frac{qV_{oc}}{m_C\,k_B\,T}\right] \qquad \text{(S24)}$$

where C_d is the dielectric capacitance (including the geometrical capacitance and further depletion layer contributions), V_d is an effective dielectric built-in voltage, C_n is the equilibrium injection/diffusion/chemical capacitance, and m_C is the capacitance ideality factor. Furthermore, from the exponential increase of capacitance in p-i-n devices a minimum doping concentration can be extracted as[44]

$$N_{d,min} = 27\frac{m_C k_B T \varepsilon_r \varepsilon_0}{4q^2L^2} \tag{S25}$$

The characteristic response times for each mechanism can be extracted via the estimation of the corresponding peak maxima across the Bode plots of the imaginary part of the impedance, and/or by considering the resistor-capacitor coupling ($R \cdot C$ product) assumed in the EC model. Either way, the behavior of the response time is as

$$\tau = \frac{\tau_0}{1 + exp\left[\frac{q(V_{oc} - V_\tau)}{m_\tau\, k_B\, T}\right]} \tag{S26}$$

where τ_0 is the equilibrium response time constant (due to dielectric dipoles and/or trap-mediated recombination), V_τ is an effective voltage threshold for radiative recombination and m_τ is a lifetime ideality factor.

S2.4.2. IS data and parameterization

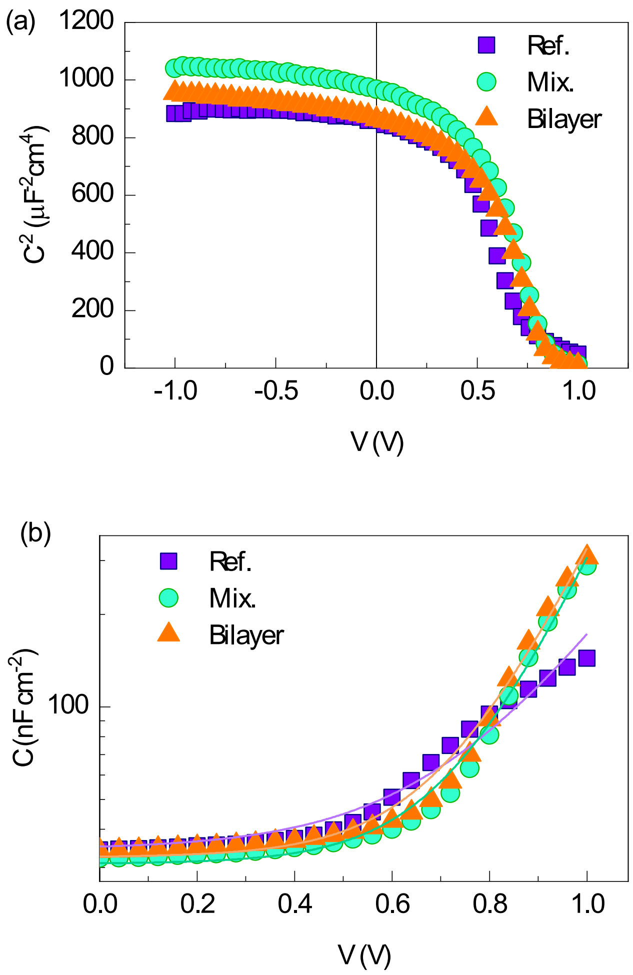

Figure S12. Dark capacitance as a function of DC bias in (a) Mott-Schottky representation and (b) semi-log plot for a set of representative studied samples, as indicated. The measurements (dots) were carried out with 20 mV of AC perturbation at 100 kHz. The solid lines in (b) are the exponential fittings to Equation (S24) whose resulting parameters are summarized in **Table S6 Table S6.** Fitted capacitance parameters

Table S6. Fitted capacitance parameters to the dark capacitance in **Figure S12**b.

Parameter	Ref.	Mix.	Bilayer
C_d (F·cm^{-2})	3.3996E-8	3.23152E-8	3.3E-8
V_d (V)	15	17.75908	5.73
C_n (F·cm^{-2})	1.47379E-10	1.5417E-11	1.96967E-11
m_C (a.u.)	4.9	3.8	3.9
$N_{d,min}$ (cm^{-3})	2.1e15	1.6e15	1.7e15

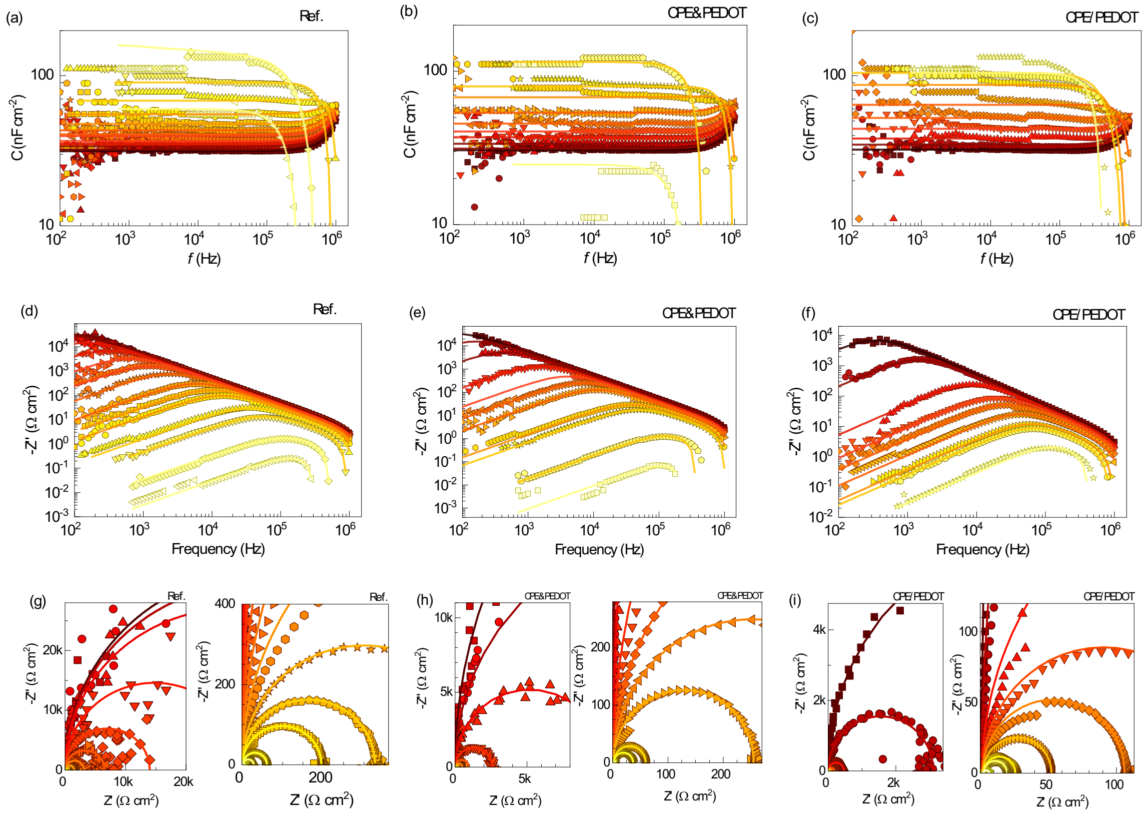

Figure S13. Impedance spectra in different representations: Bode plots of (a-c) capacitance (d-f) imaginary pary of impedance and (g-i) impedance Nyquist plots for the (a, d, g) reference, (b, e, h) CPE&PEDOT and (c, f, i) CPE/PEDOT samples.

Table S7. Fitted parameters for the EC simulation of IS spectra from the Reference sample.

V_{oc} (V)	L_s (H)	R_s (Ω)	R_1 (Ω)	C_1 (F)
0.83	3.7822E-6	16.37	14.92	3.9726E-8
0.8	5.7E-6	16.55	48.5	1.9E-8
0.75	3.2817E-6	19.73	299.9	9.259E-9
0.7	2.9E-6	17.5	663.5	6.46E-9
0.65	3.0625E-6	16.41	2151	5.048E-9
0.6	3.1771E-6	14.72	3614	4.328E-9
0.55	3.2542E-6	13.64	6614	3.9E-9
0.5	3.2351E-6	13.13	16747	3.675E-9
0.45	3.2416E-6	12.06	34743	3.564E-9
0.4	3.405E-6	11.92	72701	3.302E-9
0.35	3.4896E-6	11.47	155420	3.184E-9
0.3	3.6914E-6	10.69	327360	3.129E-9
0.25	3.5119E-6	10.76	602980	3.038E-9
0.2	4.1481E-6	10.34	698470	2.891E-9
0.1	3.6305E-6	10.28	779980	2.894E-9
0	3.6798E-6	10.03	748950	2.828E-9

Table S8. Fitted parameters for the EC simulation of IS spectra from the Mix. (CPE-Na:PEDOT:PSS) sample.

V_{oc} (V)	L_s (H)	R_s (Ω)	R_1 (Ω)	C_1 (F)
0.83	3.7665E-6	16.05	10.9	4.5217E-8
0.8	6.5E-6	16.5	39.5	2.5E-8
0.75	3.0946E-6	19.94	390.9	7.926E-9
0.7	2.9519E-6	18.03	644.7	6.449E-9
0.65	3.0539E-6	16.71	2817	4.888E-9
0.6	3.1587E-6	14.51	5553	4.117E-9
0.55	3.3219E-6	13.21	10617	3.65E-9
0.5	3.3586E-6	12.56	28640	3.338E-9
0.4	3.5035E-6	11.35	115450	3.026E-9
0.3	3.8919E-6	10.58	343880	2.812E-9
0.2	3.7321E-6	10.29	737530	2.742E-9

Table S9. Fitted parameters for the EC simulation of IS spectra from the Bilayer (CPE-Na/PEDOT:PSS) sample.

V_{oc} (V)	L_s (H)	R_s (Ω)	R_1 (Ω)	C_1 (F)
0.83	7E-6	17.0	57.0	1.8E-8
0.80	2.9046E-6	24.58	194.3	1.2E-8
0.75	2.9489E-6	22.91	263.2	9.293E-9
0.70	2.6714E-6	20.23	560.5	6.198E-9
0.65	2.7355E-6	19.14	1158	4.86E-9
0.60	2.94E-6	17.25	1979	4.052E-9
0.50	2.8832E-6	15.92	5166	3.454E-9
0.40	3.0499E-6	13.89	34413	3.121E-9
0.30	3.0767E-6	13.1	151240	2.892E-9

Table S10. Parameterization of photovoltage trend from the EC modelling of the Reference sample.

Parameters	R_s	R_1	C_1	τ_1
R_{s0} ($\Omega\cdot cm^2$)	0.656	—	—	—
V_b (V)	0.767	—	—	—
R_{sh} ($\Omega\cdot cm^2$)	—	6.56E+4	—	—
V_r (V)	—	0.260	—	—
m (a.u.)	—	2.30	—	—
C_d ($F\cdot cm^{-2}$)	—	—	3.50E-8	—
V_d (V)	—	—	2.00	—
C_n ($F\cdot cm^{-2}$)	—	—	3.46E-14	—
m_C (a.u.)	—	—	1.98	—
τ_0 (s)	—	—	—	0.00227
V_τ (V)	—	—	—	0.302
m_τ (a.u.)	—	—	—	2.40

Table S11. Parameterization of photovoltage trend from the EC modelling of the Mix. (CPE-Na:PEDOT:PSS) sample.

Parameters	R_s	R_1	C_1	τ_1
R_{s0} ($\Omega \cdot cm^2$)	0.524	—	—	—
V_b (V)	0.491	—	—	—
R_{sh} ($\Omega \cdot cm^2$)	—	7.88E+4	—	—
V_r (V)	—	0.266	—	—
m (a.u.)	—	2.4	—	—
C_d ($F \cdot cm^{-2}$)	—	—	3.17E-8	—
V_d (V)	—	—	1.5	—
C_n ($F \cdot cm^{-2}$)	—	—	3.84E-14	—
m_C (a.u.)	—	—	1.97	—
τ_0 (s)	—	—	—	0.00272
V_τ (V)	—	—	—	0.28
m_τ (a.u.)	—	—	—	2.5

Table S12. Parameterization of photovoltage trend from the EC modelling of the Bilayer (CPE-Na:PEDOT:PSS) sample.

Parameters	R_s	R_1	C_1	τ_1
R_{s0} ($\Omega \cdot cm^2$)	0.601	—	—	—
V_b (V)	0.451	—	—	—
R_{sh} ($\Omega \cdot cm^2$)	—	1.49E+4	—	—
V_r (V)	—	0.365	—	—
m (a.u.)	—	2.25	—	—
C_d ($F \cdot cm^{-2}$)	—	—	1.36E-8	—
V_d (V)	—	—	0.20	—
C_n ($F \cdot cm^{-2}$)	—	—	3.26E-14	—
m_C (a.u.)	—	—	2.10	—
τ_0 (s)	—	—	—	0.00582
V_τ (V)	—	—	—	0.10
m_τ (a.u.)	—	—	—	3.10

Ideality Factor measurements :

The use of different procedures for estimating the ideality factor focuses on contrasting the limitation of each method and provide generality to the conclusions. Table S13 summarizes the focus on recombination mechanisms, effects due to parasitic resistances, main criticisms and further relevant advantages for the employed protocols.

Table S13: Comparison between measurement methods for the ideality factor of solar cells

Method	Nonradiative recombination focus	Radiative recombination focus	Parasitic resistance effects	Main criticisms	Further relevant advantages
Dark J-V curve	For $V<<V_{bi}$	For high injection condition (e.g. $V>>V_{bi}$)	The higher the voltage the higher the effects	Lack of representativeness for photovoltaic applications	Simplicity
J_{sc}-V_{oc} under different illumination intensities	Low illumination intensities	High illumination intensities	The higher the illuminati on intensity the higher the effects	Disconnection with the fill factor and the photovoltaic operational conditions. Particularly, the photocurrent in short-circuit does not necessarily reflect the recombination mechanisms	Simplicity
IS in quasi-open circuit under different illumination intensities	Low illumination intensities	High illumination intensities	Equivalen t circuit analyses can neglect these effects	Physical validation of the equivalent circuit model.	Several recombination mechanisms can be discerned simultaneously (e.g. two arcs in the Nyquist plot)

S2.5. Stability test

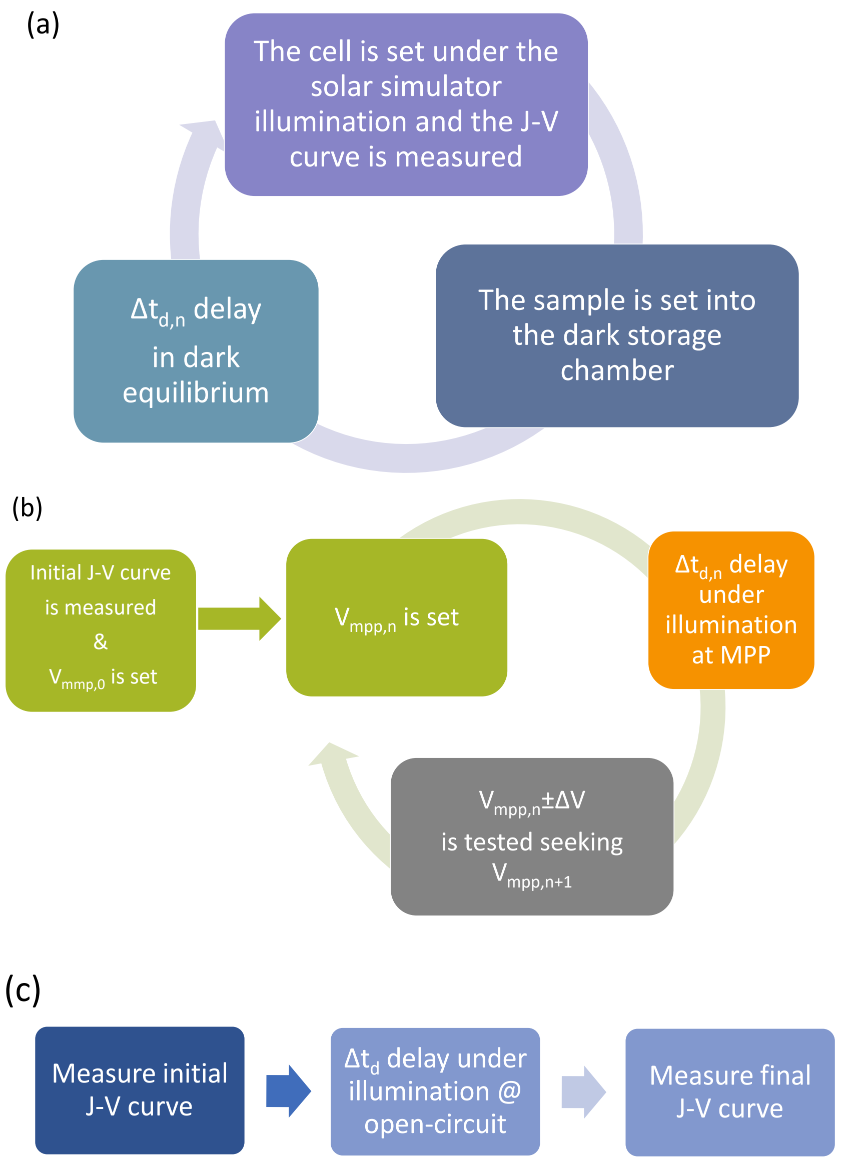

Figure S14. Device degradation test work flows for (a) dark storage and (b) maximum power point tracking operational conditions and (c) light stability test at open-circuit. Here, *Vmpp* is the voltage for the maximum power point in the current density-voltage (*J-V*) curve; ΔV, the voltage testing range; Δt_d, the degradation delay time and 0, n denote the initial and n^{th} iterations, respectively.

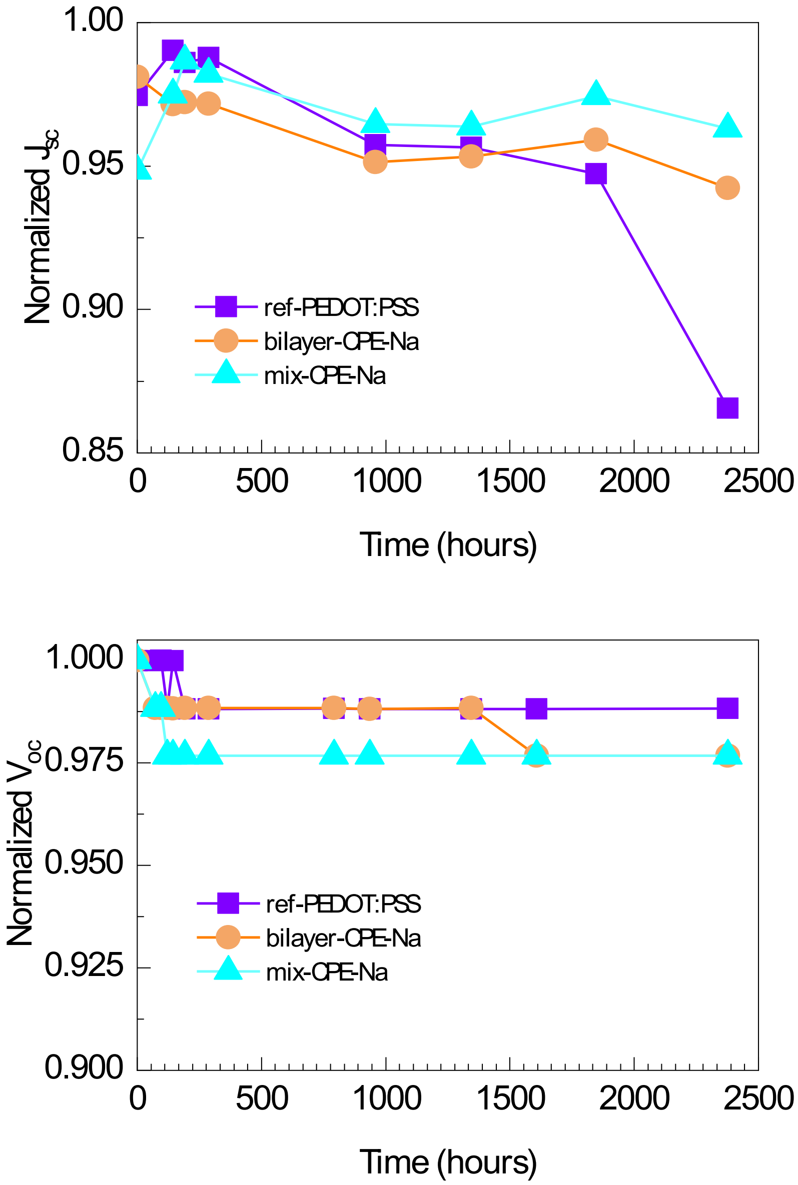

Figure S15. Device degradation tests results in conditions of dark storage attending to(a) J_{sc} (b) V_{oc}

Author Information

Corresponding Authors

Lluis F. Marsal— Department of Electronic, Electric and Automatic Engineering, Universitat Rovira i Virgili, 43007 Tarragona, Spain; https://orcid.org/0000-0002-5976-1408; Email: lluis.marsal@urv.cat

Authors

Mohamed Samir — Department of Electronic, Electric and Automatic Engineering, Universitat Rovira i Virgili, 43007 Tarragona, Spain; https://orcid.org/0000-0002-9827-1268 ; Email: mohamed.samir@urv.cat

Osbel Almora — Department of Electronic, Electric and Automatic Engineering, Universitat Rovira i Virgili, 43007 Tarragona, Spain; https://orcid.org/0000-0002-2523-0203 ; Email: osbel.almora@urv.cat

Enas Moustafa—Science and Engineering of Renewable Energy Department, Faculty of Postgraduate Studies for Advanced Science, Beni Suef University, Beni Suef, Egypt. ORCID: 0000-0003-4190-8456; Email: enas.moustafa@urv.cat

Magaly Ramírez-Como — Sección de Estudios de Posgrado e Investigación, UPIITA Instituto Politécnico Nacional, Mexico City, Mexico; ORCID: 0000-0002-9313-8337; Email: magaly.ramirez@urv.cat

Maria Pilar Montero-Rama— Department of Electronic, Electric and Automatic Engineering, Universitat Rovira i Virgili, 43007 Tarragona, Spain; Orcid: 0000-0002-4430-8723; Email: mariadelpilar.montero@urv.cat

Josep Pallarès— Department of Electronic, Electric and Automatic Engineering, Universitat Rovira i Virgili, 43007 Tarragona, Spain. Email: josep.pallares@urv.cat

José G. Sánchez — Institute of Chemical Research of Catalonia-The Barcelona Institute of Science and Technology (ICIQ-BIST), 43007 Tarragona, Spain; https://orcid.org/0000-0002-2755-8878 ; Email: jgsanchez@iciq.es

Emilio Palomares — Institute of Chemical Research of Catalonia-The Barcelona Institute of Science and Technology (ICIQ-BIST), 43007 Tarragona, Spain; https://orcid.org/0000-0002-5092-9227 ; Email: epalomares@iciq.es

Notes

The authors declare no competing financial interests.

References

(1) Oh, W.-K.; Hussain, S.Q.; Lee, Y.-J.; Lee, Y.; Ahn, S.; Yi, J. Study on the ITO work function and hole injection barrier at the interface of ITO/a-Si:H(p) in amorphous/crystalline silicon heterojunction solar cells, *Mater. Res. Bull.* **2012**, *47* (10), 3032-3035.
(2) Park, Y.; Choong, V.; Gao, Y.; Hsieh, B.R.; Tang, C.W. Work function of indium tin oxide transparent conductor measured by photoelectron spectroscopy, *Applied Physics Letters* **1996**, *68* (19), 2699-2701.
(3) Sugiyama, K.; Ishii, H.; Ouchi, Y.; Seki, K. Dependence of indium–tin–oxide work function on surface cleaning method as studied by ultraviolet and x-ray photoemission spectroscopies, *J. Appl. Phys.* **2000**, *87* (1), 295-298.
(4) Moustafa, E.; Méndez, M.; Sánchez, J.G.; Pallarès, J.; Palomares, E.; Marsal, L.F. Thermal Activation of PEDOT:PSS/PM6:Y7 Based Films Leads to Unprecedent High Short-Circuit Current Density in Nonfullerene Organic Photovoltaics, *Adv. Energy Mater.* **2023**, *13* (4), 2203241.
(5) Li, K.; Wu, Y.; Li, X.; Fu, H.; Zhan, C. 17.1%-Efficiency organic photovoltaic cell enabled with two higher-LUMO-level acceptor guests as the quaternary strategy, *Science China Chemistry* **2020**, *63* (4), 490-496.
(6) Chelvayohan, M.; Mee, C.H.B. Work function measurements on (110), (100) and (111) surfaces of silver, *J. Phys. C: Solid State Phys.* **1982**, *15* (10), 2305.
(7) Dweydari, A.W.; Mee, C.H.B. Work function measurements on (100) and (110) surfaces of silver, *Phys. Status Solidi A* **1975**, *27* (1), 223-230.
(8) Mitchell, E.W.J.; Mitchell, J.W.; Mott, N.F. The work functions of copper, silver and aluminium, *Proc. R. Soc. A* **1997**, *210* (1100), 70-84.
(9) Li, D.; Wang, L.; Guo, C.; Liu, Y.; Zhou, B.; Fu, Y.; Zhou, J.; Liu, D.; Li, W.; Wang, T. Co-crystallization of Fibrillar Polymer Donors for Efficient Ternary Organic Solar Cells, *ACS Materials Letters* **2023**, *5* (8), 2065-2073.
(10) Torimtubun, A.A.A.; Méndez, M.; Moustafa, E.; Pallarès, J.; Palomares, E.; Marsal, L.F. Achieving 17.7% Efficiency of Ternary Organic Solar Cells by Incorporating a High Lowest Unoccupied Molecular Orbital Level and Miscible Third Component, *Solar RRL* **2023**, *7* (11), 2300228.
(11) Zhang, K.-N.; Jiang, Z.-N.; Wang, T.; Niu, M.-S.; Feng, L.; Qin, C.-C.; So, S.-K.; Hao, X.-T. High-Performance Ternary Organic Solar Cells with Morphology-Modulated Hole Transfer and Improved Ultraviolet Photostability, *Solar RRL* **2020**, *4* (7), 2000165.
(12) Xu, X.; Yu, L.; Meng, H.; Dai, L.; Yan, H.; Li, R.; Peng, Q. Polymer Solar Cells with 18.74% Efficiency: From Bulk Heterojunction to Interdigitated Bulk Heterojunction, *Advanced Functional Materials* **2022**, *32* (4), 2108797.
(13) Jung, S.; Choi, S.; Shin, W.; Oh, H.; Oh, J.; Ryu, M.-Y.; Kim, W.; Park, S.; Lee, H. in *Enhancement in Power Conversion Efficiency of Perovskite Solar Cells by Reduced Non-Radiative Recombination Using a Brij C10-Mixed PEDOT:PSS Hole Transport Layer*, *Vol. 15* **2023**.
(14) Gokulnath, T.; Reddy, S.S.; Park, H.-Y.; Kim, J.; Kim, J.; Song, M.; Yoon, J.; Jin, S.-H. Nonhalogenated Solvent-Processed Thick-Film Ternary Nonfullerene Organic Solar Cells with Power Conversion Efficiency >13% Enabled by a New Wide-Bandgap Polymer, *Solar RRL* **2021**, *5* (3), 2000787.
(15) Shockley, W., *Electrons and Holes in Semiconductors with Applications to Transistor Electronics*, D. Van Nostrand, Princenton, New Jersey, **1950**, p.
(16) Shockley, W. The Theory of p-n Junctions in Semiconductors and p-n Junction Transistors, *Bell Syst. Tech. J.* **1949**, *28* (3), 435-489.

(17) Shockley, W. Electrons, Holes, and Traps, *Proceedings of the IRE* **1958**, *46* (6), 973-990.
(18) Shockley, W.; Read, W.T. Statistics of the Recombinations of Holes and Electrons, *Phys. Rev.* **1952**, *87* (5), 835-842.
(19) Sze, S.M.; Ng, K.K., *Physics of Semiconductor Devices*, John Wiley & Sons, Hoboken, New Jersey, USA, **2007**, p. 832.
(20) Tress, W.; Yavari, M.; Domanski, K.; Yadav, P.; Niesen, B.; Correa Baena, J.P.; Hagfeldt, A.; Graetzel, M. Interpretation and Evolution of Open-Circuit Voltage, Recombination, Ideality Factor and Subgap Defect States during Reversible Light-Soaking and Irreversible Degradation of Perovskite Solar Cells, *Energy Environ. Sci.* **2017**, *11* (1), 151-165.
(21) Otaegi, A.; Fano, V.; Azkona, N.; Gutiérrez, J.R.; Jimeno, J.C., On the Different Explanations of the Recombination Currents with High Ideality Factor in Silicon Solar Cells, *2017 IEEE 44th Photovoltaic Specialist Conference (PVSC)* **2017**, pp. 2740-2743.
(22) Xiong, C.; Sun, J.; Yang, H.; Jiang, H. Real reason for high ideality factor in organic solar cells: Energy disorder, *Solar Energy* **2019**, *178* 193-200.
(23) Wang, C.-X.; Yang, G.-W.; Liu, H.-W.; Han, Y.-H.; Luo, J.-F.; Gao, C.-X.; Zou, G.-T. Experimental analysis and theoretical model for anomalously high ideality factors in ZnO/diamond p-n junction diode, *App. Phys. Lett.* **2004**, *84* (13), 2427-2429.
(24) Shah, J.M.; Li, Y.-L.; Gessmann, T.; Schubert, E.F. Experimental analysis and theoretical model for anomalously high ideality factors ($n \gg 2.0$) in AlGaN/GaN p-n junction diodes, *J. Appl. Phys.* **2003**, *94* (4), 2627-2630.
(25) Liao, Y.; Li, D.; Guo, Q.; Liu, Y.; Wang, H.; Hu, W.; Wang, Z.L. Temperature-dependent study on AlGaN-based deep ultraviolet light-emitting diode for the origin of high ideality factor, *AIP Adv.* **2021**, *11* (10).
(26) Zhu, D.; Xu, J.; Noemaun, A.N.; Kim, J.K.; Schubert, E.F.; Crawford, M.H.; Koleske, D.D. The origin of the high diode-ideality factors in GaInN/GaN multiple quantum well light-emitting diodes, *App. Phys. Lett.* **2009**, *94* (8).
(27) Rana, A.; Kumar, A.; Rahman, M.W.; Vashistha, N.; Garg, K.K.; Pandey, S.; Sahoo, N.G.; Chand, S.; Singh, R.K. Non-approximated series resistance evaluation by considering high ideality factor in organic solar cell, *AIP Adv.* **2018**, *8* (12).
(28) Hadj Belgacem, C.; El-Amine, A.A. Theoretical Models for Anomalously High Ideality Factor in a Au/SnO2-Si(n)/Al Solar Cell, *Silicon* **2018**, *10* (3), 1063-1066.
(29) Lee, K.B.; Parbrook, P.J.; Wang, T.; Bai, J.; Ranalli, F.; Airey, R.J.; Hill, G. The origin of the high ideality factor in AlGaN-based quantum well ultraviolet light emitting diodes, *Phys. Status Solidi B* **2010**, *247* (7), 1761-1763.
(30) Breitenstein, O.; Altermatt, P.; Ramspeck, K.; Green, M.A.; Zhao, J.; Schenk, A., Interpretation of the Commonly Observed I-V Characteristics of C-SI Cells Having Ideality Factor Larger Than Two, *2006 IEEE 4th World Conference on Photovoltaic Energy Conference* **2006**, pp. 879-884.
(31) Tress, W. Perovskite Solar Cells on the Way to Their Radiative Efficiency Limit – Insights Into a Success Story of High Open-Circuit Voltage and Low Recombination, *Adv. Energy Mater.* **2017**, *7* (14), 1602358.
(32) Hyun Kim, C.; Yaghmazadeh, O.; Bonnassieux, Y.; Horowitz, G. Modeling the low-voltage regime of organic diodes: Origin of the ideality factor, *J. Appl. Phys.* **2011**, *110* (9).
(33) Sozzi, G.; Mosca, R.; Calicchio, M.; Menozzi, R., Anomalous dark current ideality factor ($n > 2$) in thin-film solar cells: The role of grain-boundary defects, *2014 IEEE 40th Photovoltaic Specialist Conference (PVSC)* **2014**, pp. 1718-1721.
(34) Almora, O. in *Hysteresis and Capacitive features of Perovskite Solar Cells*, *Vol. PhD* Friedrich-Alexander Universität Erlangen-Nürnberg & Universitat Jaume I Erlangen, **2020**.
(35) Bashahu, M.; Nkundabakura, P. Review and tests of methods for the determination of the solar cell junction ideality factors, *Solar Energy* **2007**, *81* (7), 856-863.

(36) Calado, P.; Burkitt, D.; Yao, J.; Troughton, J.; Watson, T.M.; Carnie, M.J.; Telford, A.M.; O'Regan, B.C.; Nelson, J.; Barnes, P.R.F. Identifying Dominant Recombination Mechanisms in Perovskite Solar Cells by Measuring the Transient Ideality Factor, *Phys. Rev. Appl.* **2019**, *11* (4), 044005.
(37) Bayhan, H.; Bayhan, M. A simple approach to determine the solar cell diode ideality factor under illumination, *Solar Energy* **2011**, *85* (5), 769-775.
(38) Masui, H.; Nakamura, S.; DenBaars, S.P. Technique to evaluate the diode ideality factor of light-emitting diodes, *Appl. Phys. Lett.* **2010**, *96* (7).
(39) Almora, O.; Matt, G.J.; These, A.; Kanak, A.; Levchuk, I.; Shrestha, S.; Osvet, A.; Brabec, C.J.; Garcia-Belmonte, G. Surface versus Bulk Currents and Ionic Space-Charge Effects in $CsPbBr_3$ Single Crystals, *J. Phys. Chem. Lett.* **2022**, *13* (17), 3824-3830.
(40) Almora, O.; Lopez-Varo, P.; Cho, K.T.; Aghazada, S.; Meng, W.; Hou, Y.; Echeverría-Arrondo, C.; Zimmermann, I.; Matt, G.J.; Jiménez-Tejada, J.A., et al. Ionic Dipolar Switching Hinders Charge Collection in Perovskite Solar Cells with Normal and Inverted Hysteresis, *Sol. Energy Mater. Sol. Cells* **2019**, *195* 291-298.
(41) Almora, O.; Cho, K.T.; Aghazada, S.; Zimmermann, I.; Matt, G.J.; Brabec, C.J.; Nazeeruddin, M.K.; Garcia-Belmonte, G. Discerning Recombination Mechanisms and Ideality Factors through Impedance Analysis of High-Efficiency Perovskite Solar Cells, *Nano Energy* **2018**, *48* 63-72.
(42) Wu, J.-L.; Chen, F.-C.; Hsiao, Y.-S.; Chien, F.-C.; Chen, P.; Kuo, C.-H.; Huang, M.H.; Hsu, C.-S. Surface Plasmonic Effects of Metallic Nanoparticles on the Performance of Polymer Bulk Heterojunction Solar Cells, *ACS Nano* **2011**, *5* (2), 959-967.
(43) Palomares, E.; Montcada, N.F.; Méndez, M.; Jiménez-López, J.; Yang, W.; Boschloo, G. in *Chapter 7 - Photovoltage/photocurrent transient techniques*, Eds.: M. Pazoki, A. Hagfeldt and T. Edvinsson), Elsevier, **2020**, pp. 161-180.
(44) Ravishankar, S.; Unold, T.; Kirchartz, T. Comment on "Resolving spatial and energetic distributions of trap states in metal halide perovskite solar cells", *Science* **2021**, *371* (6532), eabd8014.
